# Differences in mitochondrial NADH dehydrogenase activities in trypanosomatids

**DOI:** 10.1017/S0031182020002425

**Published:** 2021-09

**Authors:** Petra Čermáková, Anna Maďarová, Peter Baráth, Jana Bellová, Vyacheslav Yurchenko, Anton Horváth

**Affiliations:** 1 Department of Biochemistry, Faculty of Natural Sciences, Comenius University, Bratislava, Slovakia; 2 Institute of Chemistry, Slovak Academy of Sciences, Bratislava, Slovakia; 3 Faculty of Science, Life Science Research Centre, University of Ostrava, Ostrava, Czech Republic; 4 Martsinovsky Institute of Medical Parasitology, Tropical and Vector Borne Diseases, Sechenov University, Moscow, Russia

**Keywords:** Monoxenous trypanosomatids, NADH dehydrogenase, *Phytomonas*

## Abstract

Complex I (NADH dehydrogenase) is the first enzyme in the respiratory chain. It catalyses the electron transfer from NADH to ubiquinone that is associated with proton pumping out of the matrix. In this study, we characterized NADH dehydrogenase activity in seven monoxenous trypanosomatid species: *Blechomonas ayalai*, *Herpetomonas tarakana*, *Kentomonas sorsogonicus*, *Leptomonas seymouri*, *Novymonas esmeraldas*, *Sergeia podlipaevi* and *Wallacemonas raviniae*. We also investigated the subunit composition of the complex I in dixenous *Phytomonas serpens*, in which its presence and activity have been previously documented. In addition to *P. serpens*, the complex I is functionally active in *N. esmeraldas* and *S. podlipaevi*. We also identified 24–32 subunits of the complex I in individual species by using mass spectrometry. Among them, for the first time, we recognized several proteins of the mitochondrial DNA origin.

## Introduction

NADH:ubiquinone oxidoreductase [EC 7.1.1.2], eukaryotic complex I, is the largest and the most complicated enzyme of the respiratory chain. Its subunits are encoded by both the nuclear and mitochondrial genomes (Chomyn *et al*., 1985; Walker *et al*., 1992). It couples the transfer of two electrons from NADH to ubiquinone to the translocation of four protons across the mitochondrial inner membrane. The proposed mechanism includes conformation changes, electrostatic interactions and water molecules that constitute proton-translocation pathways (Grba and Hirst, 2020; Kampjut and Sazanov, 2020). Complex I has an L-shaped structure with a hydrophilic peripheral and a hydrophobic membrane domain. The hydrophilic arm contains two enzymatically distinct regions: the N-module involved in the oxidation of NADH and subsequent electron transport, forming a tip of the arm, and the Q-module, which contains Fe–S clusters, through which electrons are transferred to ubiquinone, forming the interface between two domains. The hydrophobic P-module (composed of the ND1, ND2, ND4 and ND5 multi-protein modules taking part in the proton pumping) is embedded in the inner mitochondrial membrane (Yagi and Matsuno-Yagi, 2003; Brandt, 2006, 2013; Berrisford and Sazanov, 2009). The core of this enzyme consists of 14 essential subunits that are fairly conserved across different domains of life (Gabaldón *et al*., 2005). Mammalian complex I additionally contains up to 32 accessory subunits that are not directly associated with energy conservation (Carroll *et al*., 2006; Kmita and Zickermann, 2013). These proteins may be involved in the regulation of enzymatic activity, stability of the complex or auxiliary functions, for example, the fatty acid synthesis (Janssen *et al*., 2006; Pereira *et al*., 2013). Two of the most commonly used inhibitors of the mitochondrial complex I are rotenone (stabilizing the semiquinone intermediate within the complex) and capsaicin (antagonizing either formation or release of the quinol product) (Degli Esposti, 1998; Okun *et al*., 1999).

In addition to complex I, another NADH dehydrogenase, NDH2, has been discovered in the mitochondria of several organisms. It catalyses the transfer of electrons from NADH to ubiquinone without pumping protons out of the matrix (Matus-Ortega *et al*., 2011). In extreme cases (for example, in *Saccharomyces cerevisiae*), the complex I is completely missing and its function is taken by the alternative dehydrogenases (Overkamp *et al*., 2000).

Trypanosomatids (class Kinetoplastea) is a group of obligate parasitic flagellates confined exclusively to insects (monoxenous species) or transmitted by insects or annelids to vertebrates or plants (Lukeš *et al*., 2018; Maslov *et al*., 2019). Functionality of the trypanosomatid complex I has long been debated. Bioinformatics analysis identified 29 orthologue genes of the prototypical eukaryotic subunits and further 34 genes encoding unique accessory proteins in genomes of dixenous *Trypanosoma brucei*, *T. cruzi* and *Leishmania major* (Opperdoes and Michels, 2008; Perez *et al*., 2014; Opperdoes *et al*., 2016). These genomic data suggest that trypanosomatid complex I is composed of over 60 subunits and its molecular mass is over 2 MDa, which is twice as large as its bovine or yeast counterpart (Abdrakhmanova *et al*., 2004; Carroll *et al*., 2006). The mitochondrial DNA of *Trypanosoma* spp. encodes eight complex I subunits. ND1–ND5 are orthologues to the mitochondrial subunits (which participate in protons pumping and bind ubiquinone and rotenone), while ND7–ND9 are orthologues to the nuclear-encoded subunits NDUFS2 (Fe–S cluster and binding site for ubiquinone), NDUFS8 (two Fe–S clusters) and NDUFS3 in humans. The genes for ND4L and ND6 had been assigned to neither the mitochondrial nor to the nuclear DNA (Opperdoes and Michels, 2008). However, it has been proposed that these proteins are encoded in the mitochondria by *CR3* and *CR4* genes (Duarte and Tomás, 2014). Recent data demonstrated that trypanosomatids possess all the proteins necessary for NAD^+^ regeneration by complex I: those involved in electron transfer, ubiquinone binding and reduction and proton pumping. Proteomic analysis confirmed the presence of both canonical and auxiliary subunits encoded in the nuclear genome of *T. brucei*. It was clearly showed that the complex I subunits are organized into the high molecular weight proteins in trypanosomal mitochondria (Panigrahi *et al*., 2008; Acestor *et al*., 2011). However, none of the proteomic studies published to date has been able to detect complex I subunits encoded by the mitochondrial genome of trypanosomatids.

The importance of the complex I in trypanosomatids has been disputed. Indeed, both dyskinetoplastic *Trypanosoma evansi* and *T. equiperdum* thrive without it (Schnaufer *et al*., 2002). The natural *T. cruzi* mutants with deletions in *ND4*, *ND5* and *ND7* genes showed no alterations in mitochondrial bioenergetics compared to the wild type (Carranza *et al*., 2009). Long-term cultivated isolates of *Leishmania tarentolae* and *Crithidia fasciculata* have lost guide RNAs for editing of *ND3*, *ND8* and *ND9* genes and no complex I activity had been detected in them (Sloof *et al*., 1994; Thiemann *et al*., 1994). Complex I is also not essential in the studied stages of *T. brucei*. Ablation of NDUFV1 and NDUFS7 in the procyclic and bloodstream forms did not produce any effect on the detected NADH dehydrogenase activity (Verner *et al*., 2011; Surve *et al*., 2012), which was also not sensitive to the rotenone (Verner *et al*., 2014). Of note, the presence of alternative NDH2 has been documented in *T. brucei* (Fang and Beattie, 2002; Verner *et al*., 2013) and *Phytomonas serpens* (Gonzalez-Halphen and Maslov, 2004; Čermáková *et al*., 2007). Its elimination in both procyclic and bloodstream forms of *T. brucei* had only a modest effect on the viability of the tested cells (Verner *et al*., 2013; Surve *et al*., 2017).

The only trypanosomatid species with essential mitochondrial complex I known to date is *P. serpens* (Čermáková *et al*., 2007). However, it lacks respiratory chain complexes III and IV (Nawathean and Maslov, 2000). The size of the complex I in that species is about 2.2 MDa and its NADH dehydrogenase activity, as well as mitochondrial membrane potential are sensitive to rotenone (Moyses and Barrabin, 2004; Verner *et al*., 2014). It was demonstrated that the complex contains subunits NDUFA6 and NDUFA9 (Čermáková *et al*., 2007).

Here, we investigated NADH dehydrogenase activity in *P. serpens* and seven monoxenous trypanosomatids: *Blechomonas ayalai* (Votýpka *et al*., 2013), *Herpetomonas tarakana* (Yurchenko *et al*., 2016), *Kentomonas sorsogonicus* (Votýpka *et al*., 2014), *Leptomonas seymouri* (Wallace, 1977), *Novymonas esmeraldas* (Kostygov *et al*., 2016), *Sergeia podlipaevi* (Svobodová *et al*., 2007) and *Wallacemonas raviniae* (Kostygov *et al*., 2014). We provide evidence that functional complex I is present in two more trypanosomatids. In these molecular complexes, we detected not only a majority of the nuclear DNA-encoded proteins, but (for the first time) also several subunits derived from the mitochondrial DNA. In all of them we also spotted MURF2, the protein of unknown function (Blum and Simpson, 1990).

## Materials and methods

### Cultivation of trypanosomatids

*Phytomonas serpens* (strain 9T) was grown at 27°C in brain heart infusion (BHI) medium (Becton, Dickinson and Co, Sparks, USA) supplemented with 10 *μ*g mL^−1^ haemin (AppliChem, Darmstadt, Germany) (Lukeš *et al*., 2006). *Herpetomonas tarakana* (strain OSR18) was cultivated at 27°C in the complete M199 medium (Sigma-Aldrich, St. Louis, USA) supplemented with 2 *μ*g mL^−1^ haemin, 10% foetal bovine serum (FBS, Biosera, Kansas City, USA), 100 U mL^−1^ penicillin, 100 *μ*g mL^−1^ streptomycin (Sigma-Aldrich), 2 *μ*g mL^−1^ biopterin (Sigma-Aldrich) and 25 mm HEPES (AppliChem). *Blechomonas ayalai* (strain B08-376), *K. sorsogonicus* (strain MF08-01), *L. seymouri* (strain ATCC30220), *N. esmeraldas* (strain E262.01), *S. podlipaevi* (strain CER3) and *W. raviniae* (strain Mbr-04) were cultured at 23°C in BHI medium supplemented with 10 *μ*g mL^−1^ haemin, 10% FBS, 100 U mL^−1^ penicillin, 100 *μ*g mL^−1^ streptomycin.

### Preparation of mitochondrial lysate

The mitochondria-enriched fractions from 5 × 10^8^ cells were isolated by hypotonic lysis as described elsewhere (Horváth *et al*., 2005). Mitochondria were re-suspended in 0.5 m aminocaproic acid and 2% (w/v) dodecyl maltoside (both AppliChem). Lysis was performed for 1 h on ice and the lysates were centrifuged for 10 min at 20 000 × ***g*** at 4°C. The supernatants were recovered, and protein concentration was determined by the Bradford assay (Bradford, 1976).

### *In silico* analyses

The genome of *T. brucei* [available from the TriTrypDB (Aslett *et al*., 2010)] was used as a template to search for genes of nucleus-encoded complex I subunits and NDH2 in other trypanosomatid genomes – *B. ayalai* (Opperdoes *et al*., 2016), *L. seymouri* (Kraeva *et al*., 2015), *N. esmeraldas* (manuscript in preparation) and *W. raviniae* (manuscript in preparation) – using BLAST v.2.6.0+ (Camacho *et al*., 2009).

### NADH dehydrogenase activity assay

NADH dehydrogenase activity was measured in 1 mL NDH buffer (50 mm potassium phosphate buffer, pH 7.5, 1 mm EDTA, 0.2 mm KCN), containing 20–30 *μ*g proteins from the mitochondrial lysates and 5 *μ*L of 20 mm NADH (AppliChem). After the addition of 2 *μ*L 10 mm coenzyme Q_2_ (Sigma-Aldrich), the change in absorbance at 340 nm was followed for 3 min (Čermáková *et al*., 2019). A unit of activity was defined as the amount of enzyme that catalyses the oxidation of 1 nmol NADH per min, assuming an extinction coefficient of 6.2 L mmol^−1^ cm^−1^ (Gonzalez-Halphen and Maslov, 2004). Solutions of the inhibitors were freshly prepared. Capsaicin (Sigma-Aldrich) was dissolved in ethanol, rotenone (Serva, Heidelberg, Germany) and DPI (diphenyl iodonium, Sigma-Aldrich) – in dimethylsulphoxide and methanol, respectively. Rotenone and DPI were added to the assay mixture immediately before the start of the reaction, capsaicin was pre-incubated for 3 min. Native electrophoresis and in-gel activity staining methods were adapted from Zerbetto *et al*. (1997) and Wittig *et al*. (2007) and performed as described previously (Verner *et al*., 2014).

### In-gel digestion and mass spectrometry analysis

Procedure was performed as previously described (Shevchenko *et al*., 2006). Briefly, proteins were separated by native gradient gel, bands of interest were cut into small pieces and incubated in 100 mm ammonium bicarbonate buffer. The samples were reduced in 10 mm DTT (30 min, 56°C) and dehydrated in acetonitrile. Alkylation reaction was performed in the presence of 15 mm iodoacetamide (20 min, room temperature, dark) and samples were dehydrated as described above. For protein digestion, 500 ng of the sequencing grade trypsin (Promega, Madison, USA) and 1 mm CaCl_2_ were added and the samples were incubated on ice for 30 min (if digestion was incomplete, the reaction was incubated overnight at 37°C). Digested peptides were eluted with acetonitrile and dried in SpeedVac (Thermo Fisher Scientific, Waltham, USA).

For liquid chromatography-mass spectroscopy (LC-MS) analysis, the set of a Nano-trap column (Acclaim PepMap100 C18, 75 *μ*m × 20 mm) and Nano-separation column (Acclaim PepMap C18, 75 *μ*m × 500 mm, both Dionex, Sunnyvale, USA/Thermo Fisher Scientific) attached to the UltiMate 3000 RSLCnano system (Dionex) was used. The peptides were separated for 120 min in a 3–43% gradient of buffer B with two mobile phases used: 0.1% formic acid (v/v) (buffer A) and 80% acetonitrile (v/v) with 0.1% formic acid (buffer B). Spectral data were collected by using the Orbitrap Elite mass spectrometer (Thermo Fisher Scientific) operating in the data-dependent mode using the Top15 strategy for the selection of precursor ions for the HCD fragmentation (Michalski *et al*., 2012). Obtained datasets were processed by MaxQuant v.1.5.3.30 with built-in Andromeda search engine (Cox *et al*., 2011). The specific parameters for searching were: carbamidomethylation (C) as permanent modification and oxidation (M) and acetyl (protein N-terminus) as variable modifications. The search was performed against protein datasets of *Phytomonas* sp. (Hart1), *T. brucei* (TREU927), *T. brucei* (Lister 427), *L. major* (Friedlin) (TriTrypDB, downloaded 10.10.2020) and against a sequence database of U insertion/deletion editing in kinetoplastid mitochondria (Simpson *et al*., 1998).

## Results

We have characterized NADH dehydrogenase activity in *P. serpens* and seven monoxenous trypanosomatids. We selected species from different clades of Trypanosomatidae (Lukeš *et al*., 2018). These are the members of the subfamilies Leishmaniinae (Kostygov and Yurchenko, 2017) (*L. seymouri* and *N. esmeraldas*), Strigomonadinae (Votýpka *et al*., 2014) (*K. sorsogonicus*), Phytomonadinae (Yurchenko *et al*., 2016) (*H. tarakana*), Blechomonadinae (Votýpka *et al*., 2013) (*B. ayalai*), as well as two genera not formally classified into any subfamily – *Sergeia* (Svobodová *et al*., 2007) and *Wallacemonas* (Kostygov *et al*., 2014). These species differ not only in host specificity (Dictyoptera, Diptera, Heteroptera or Siphonaptera), but also geographical distribution and particulars of their life cycle. *Novymonas esmeraldas* and *K. sorsogonicus* harbour endosymbiotic bacteria, which have been acquired by host species independently in evolution (Kostygov *et al*., 2017; Silva *et al*., 2018), while *L. seymouri* and *B. ayalai* are heavily infected with dsRNA viruses (Grybchuk *et al*., 2018*a*, 2018*b*).

### *In silico* analyses

We examined the presence of 19 core subunits of both the membrane and peripheral domains of the complex I, whose human orthologues were identified in *T. brucei* (Duarte and Tomás, 2014), and an alternative pathway enzyme, NDH2, in analysed species of trypanosomatids. The genomic data were available only for four species. The genomes of *B. ayalai* and *L. seymouri* are in TriTrypDB and two genomes were sequenced by us: *N. esmeraldas* (32 Mbp; N50 197 811 bp; 1422 scaffolds) and *W. raviniae* (27 Mbp; N50 58 925; 1386 scaffolds) (both unpublished data). The correspondent sequences of *T. brucei* TREU927 from the TriTrypDB (Aslett *et al*., 2010) were used as queries to search the *N. esmeraldas* and *W. raviniae* assemblies with TBLASTN+ v.2.6.0 (Camacho *et al*., 2009) using a threshold of 10^−50^. The obtained hits were reciprocally BLASTed against the NCBI database. In all the cases, the genes of interest were located in syntenic genomic positions. All tested genes were detected in the genomes of all analysed trypanosomatids (Table 1 and Supplementary Table 1). Of note, multiple copies for genes encoding subunits NDUFA8, NDUFB10 and NDUFA12 were documented in the genome of *W. raviniae*.

**Table 1.** *In silico* analysis of the selected complex I genes and alternative dehydrogenase NDH2 encoded by nuclear DNA

Species	*Homo sapiens*	*Trypanosoma brucei*	*Blechomonas ayalai*	*Leptomonas seymouri*	*Novymonas esmeraldas*	*Wallacemonas raviniae*
Membrane domain	NDUFB1	Tb927.11.7390	Baya_011_0530	Lsey_0055_0260	+	+
	NDUFB7	Tb927.9.11660	Baya_100_0220	Lsey_0192_0100	+	+
	NDUFB9	Tb927.11.15810	Baya_019_0320	Lsey_0010_0080	+	+
	NDUFB10	Tb927.11.9930	Baya_039_0260	Lsey_0091_0010	+	+ (2)
	NDUFB11	Tb927.4.440	Baya_165_0080	Lsey_0525_0020	+	+
	NDUFAB1	Tb927.3.860	Baya_111_0040	Lsey_0115_0040	+	+
	NDUFS5	Tb927.3.5340	Baya_092_0110	Lsey_0041_0050	+	+
	NDUFA6	Tb927.10.14860	Baya_244_0010	Lsey_0011_0010	+	+
	NDUFA8	Tb927.10.12930	Baya_093_0130	Lsey_0013_0050	+	+ (3)
	NDUFA9	Tb927.10.13620	Baya_084_0070	Lsey_0157_0100	+	+
Peripheral domain	NDUFA13	Tb927.11.8910	Baya_029_0060	Lsey_0071_0190	+	+
	NDUFA12	Tb927.9.12680	Baya_004_0460	Lsey_0186_0050	+	+ (2)
	NDUFA5	Tb927.10.4130	Baya_191_0090	Lsey_0122_0100	+	+
	NDUFA2	Tb927.11.16870	Baya_038_0390	Lsey_0241_0060	+	+
	NDUFS7	Tb927.11.1320	Baya_018_0020	Lsey_0065_0230	+	+
	NDUFS6	Tb927.6.4270	Baya_060_0270	Lsey_0209_0010	+	+
	NDUFS1	Tb927.10.12540	Baya_080_0190	Lsey_0113_0150	+	+
	NDUFV2	Tb927.7.6350	Baya_155_0060	Lsey_0197_0040	+	+
	NDUFV1	Tb927.5.450	Baya_008_1080	Lsey_0248_0020	+	+
NDH2	–	Tb927.10.9440	Baya_062_0020	Lsey_0004_0940	+	+

All selected genes were detected in all analysed trypanosomatid genomes. The table lists either the names of genes in the TriTrypDB that was used for *T. brucei*, *B. ayalai* and *L. seymouri* or the ‘**+’** sign indicating the presence in unannotated databases for *N. esmeraldas* and *W. raviniae*. All genes were found in one copy, except for a few genes of *W. raviniae*, for which a higher copy number is given in parentheses. Names of *H. sapiens* orthologues are also provided.

#### NADH dehydrogenase activity

**Fig. 1.**
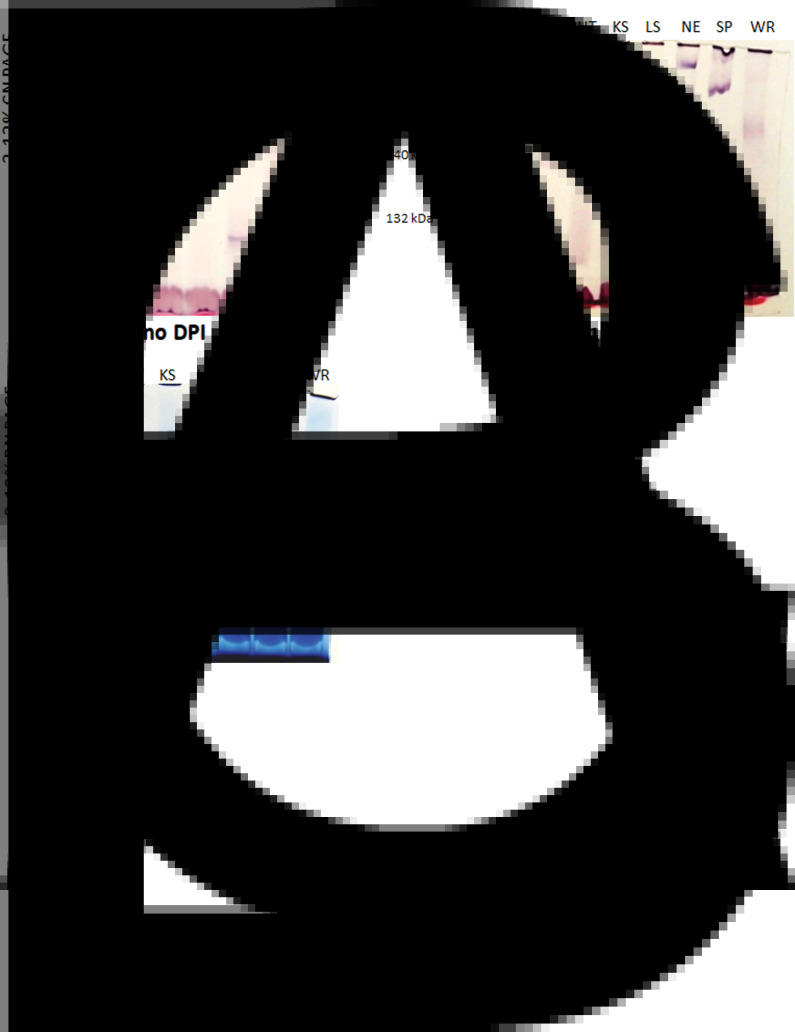
In-gel NADH dehydrogenase activity staining. (A, B) Clear native and (C) blue native gradient gel; 100 *μ*g of mitochondrial proteins from *Phytomonas serpens* (PS), *Blechomonas ayalai* (BA), *Herpetomonas tarakana* (HT), *Kentomonas sorsogonicus* (KS), *Leptomonas seymouri* (LS), *Novymonas esmeraldas* (NE), *Sergeia podlipaevi* (SP) and *Wallacemonas raviniae* (WR) were applied to each lane. The NADH dehydrogenase activity was detected without (A, C) or with (B) 100 *μ*m DPI. The slices with NADH dehydrogenase activity from blue native gel (C) subjected to MS analysis are marked by numbers 1–4. The positions of molecular weight markers (dimer of BSA and monomer, dimer and trimer of ferritin) are indicated.

Mitochondrial proteins of the studied strains were separated in 2–12% clear native gradient gel and NADH dehydrogenase activity was detected by in-gel staining (Fig. 1A). In the high molecular weight range, we detected NADH dehydrogenase activity in all species tested. However, the intensity and number of active bands differed significantly. We also noticed significant differences when comparing two different types of native electrophoresis – clear native (Fig. 1A) and blue native (Fig. 1C). NADH dehydrogenase activity in the low molecular weight range was observed for *K. sorsogonicus* and *N. esmeraldas*. Its molecular weight around 130 kDa could correspond to the NDH2 dimer. For distinguishing different NADH dehydrogenase activities we performed in-gel staining in the presence of 100 *μ*m DPI (Fig. 1B), which inhibits NDH2 and incomplete complex I (Čermáková *et al*., 2007). In the case of analysed trypanosomatids, DPI has inhibited most of the signals – strong bands remained visible only in the samples of *P. serpens*, *N. esmeraldas* and *S. podlipaevi*. This suggests that DPI-resistant activity in *N. esmeraldas* and *S. podlipaevi* corresponds to the complex I, as was previously shown in *P. serpen*s (Čermáková *et al*., 2007).

NADH dehydrogenase activity was spectrophotometrically measured in four trypanosomatid species (selected based on either the strongest intensity of in-gel staining signal or the presence of activity in low molecular weight range – *N. esmeraldas*, *S. podlipaevi*, *W. raviniae* and *K*. *sorsogonicus*) in the absence or presence of specific inhibitors of the eukaryotic complex I (rotenone and capsaicin) and DPI. Our data demonstrated that contribution of the complex I and NDH2 is about equal in *P. serpens* (Table 2). Although the inhibitory effect of rotenone and capsaicin was comparable in this species, in all other trypanosomatids rotenone did not inhibit NADH dehydrogenase activity. In addition to *P. serpens*, capsaicin inhibited NADH dehydrogenase activity in *N. esmeraldas* and *S. podlipaevi*. A comparable degree of inhibition by capsaicin and DPI in all three species implies the presence of both the functional complex I and the alternative NDH2. *Kentomonas sorsogonicus* and *W. raviniae* were not sensitive to capsaicin, while sensitive to DPI, which inhibited over 80% NADH dehydrogenase activity in the *W. raviniae* and blocked it completely in *K. sorsogonicus* (Table 2). These results correlate with DPI sensitivity of NADH dehydrogenase determined in the gel (Fig. 1B) and do not indicate the presence of a fully functional complex I in the tested life stage of both *W. raviniae* and *K. sorsogonicus*.

**Table 2.** Specific NADH dehydrogenase activity with and without inhibitors

Species	Specific activity (U mg^−1^)	Inhibitor	Inhibition (%)
*Phytomonas serpens*	28 ± 11	Rotenone	30 ± 4
		Capsaicin	42 ± 6
		DPI	37 ± 4
*Kentomonas sorsogonicus*	39 ± 8	Rotenone	2 ± 3
		Capsaicin	9 ± 8
		DPI	100 ± 0
*N. esmeraldas*	20 ± 9	Rotenone	9 ± 7
		Capsaicin	34 ± 12
		DPI	35 ± 8
*Sergeia podlipaevi*	27 ± 10	Rotenone	6 ± 3
		Capsaicin	27 ± 9
		DPI	20 ± 4
*W. raviniae*	110 ± 24	Rotenone	7 ± 4
		Capsaicin	8 ± 2
		DPI	81 ± 13

NADH dehydrogenase activity was measured in the mitochondrial lysates of *P. serpens*, *K. sorsogonicus*, *N. esmeraldas*, *S. podlipaevi* and *W. raviniae* in the absence or presence of 10 *μ*m rotenone, 300 *μ*m capsaicin and 100 *μ*m DPI. Average values and s.d. of activities and their inhibition (in %) from at least three independent biological replicated (each measured in triplicates) are presented. One unit (U) of NADH dehydrogenase activity catalyses the oxidation of 1 nmol NADH per minute. Specific activity is calculated as U mg^−1^ of mitochondrial proteins.

#### Protein composition of the NADH dehydrogenase complex

**Fig. 2.**
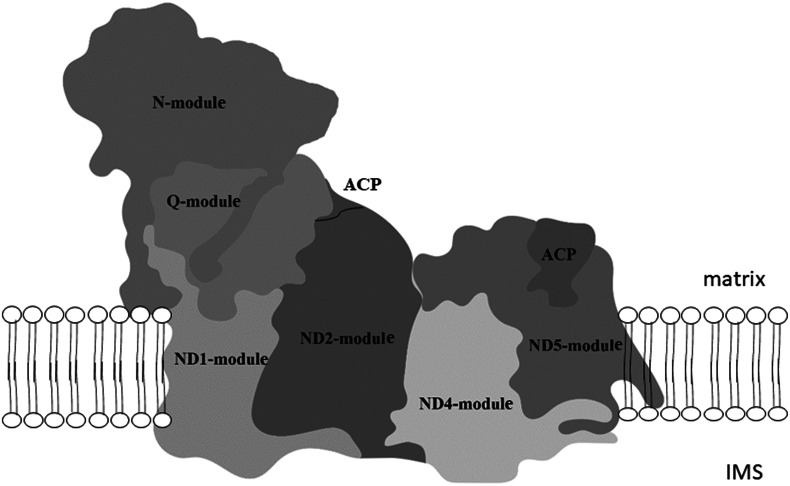
Modular composition of the complex I. The different modules: N-module, Q-module, P- module (composed of ND1, ND2, ND4 and ND5) and acyl carrier protein (ACP) are shown superimposing the structure of bovine complex I. The matrix and intermembrane space (IMS) site of inner mitochondrial membrane are indicated. Adapted from Stroud *et al*. (2016).

Four native gel's strips in the high molecular weight range of *P. serpens*, *N. esmeraldas* and *S. podlipaevi* and around 130 kDa of *N. esmeraldas* (Fig. 1C) were subjected to LC-MS analysis (Supplementary Table 2). Most of the returned hits were hypothetical proteins, yet we were able to identify 29 nuclear-encoded subunits of the *P. serpens* complex I and 22 and 23 subunits of this complex in *N. esmeraldas* and *S. podlipaevi* datasets, respectively (Table 3). All the identified subunits were localized to the complex I modules: the N-module forming the peripheral arm, the Q-module binding the ubiquinone, and ND1, ND4 ND5 modules forming the membrane part of the complex I. The only part, from which no subunit has been identified, is the ND2 module. The acyl-carrier protein NDUFAB1 (that is not part of any module) was also detected (Fig. 2, Table 3). In addition to the subunits orthologues to those in other organisms, we also recognized some additional trypanosomatid-specific complex I subunits (Duarte and Tomás, 2014) and a few other proteins that are annotated in the TriTrypDB as NADH dehydrogenase subunits without detailed specification. In addition to the nuclear DNA-encoded complex I subunits, we have also detected several proteins encoded in mitochondrial DNA: ND8, ND7 and ND1. To the best of our knowledge, this is the first experimental detection of mitochondrial DNA-encoded subunits of the complex I at the protein level in trypanosomatids. Moreover, our analysis revealed the presence of the MURF2 (mitochondrial protein with unknown function) in a high molecular weight signals range of NADH dehydrogenase activity. Its detection in all three examined species suggests that this protein may be a part of the trypanosomatids complex I.

**Table 3.** Subunits of mitochondrial complex I detected by mass spectrometry analysis

	*P. serpens*	*N. esmeraldas*	*S. podlipaevi*
N-module	NDUFV1	NDUFV1	NDUFV1
	NDUFA12	NDUFA12	NDUFA12
	NDUFV2	NDUFV2	
	NDUFA2	NDUFA2	NDUFA2
	NDUFS1	NDUFS1	NDUFS1
	NDUFA6	NDUFA6	NDUFA6
Q-module	NDUFA5	NDUFA5	NDUFA5
	NDUFS7	NDUFS7	NDUFS7
	NDUFA9		NDUFA9
	NDUFS8 (ND8*)	NDUFS8 (ND8*)	NDUFS8 (ND8*)
			NDUFS2 (ND7*)
ND1-module	NDUFA8	NDUFA8	NDUFA8
	NDUFA13	NDUFA13	NDUFA13
	ND1*		ND1*
ND4-module	NDUFB11	NDUFB11	NDUFB11
	NDUFB10	NDUFB10	NDUFB10
	NDUFB1	NDUFB1	
ND5-module	NDUFB7	NDUFB7	
		NDUFB9	NDUFB9
Acyl carrier protein (ACP)	NDUFAB1	NDUFAB1	NDUFAB1
Unique trypanosomatids accessory subunits	NDUTB2		NDUTB2
	NDUTB3		
	NDUTB5	NDUTB5	NDUTB5
	NDUTB10		NDUTB10
			NDUTB11
	NDUTB12	NDUTB12	NDUTB12
	NDUTB15	NDUTB15	NDUTB15
	NDUTB17		NDUTB17
	NDUTB25		
	NDUTB26	NDUTB26	NDUTB26
	NDUTB31	NDUTB31	
Others	Tb927.10.5500	Tb927.10.5500	Tb927.10.5500
	Tb927.11.7212		
	Tb927.11.15440		
	MURF2*	MURF2*	MURF2*
Total	32	24	27

Distribution of identified subunits to the modules of complex I is indicated in the left column. Designation of *H. sapiens* subunits in modules and ACP rows and *T. brucei* subunits in other rows were used. Subunits encoded by mitochondrial DNA are marked with * (ND1, ND7, ND8 and MURF2).

In addition to the complex I proteins, we also identified nine subunits of the ATP synthase, both alternative oxidases (orthologues of Tb927.10.7090 and Tb927.10.9760) and one component of the 2-oxoglutarate dehydrogenase complex (orthologue of Tb927.11.16730) in *P. serpens*; eight subunits of the ATP synthase, six subunits of the cytochrome *c* oxidase including mitochondrial DNA-encoded COII and COIII, three subunits of the cytochrome *c* reductase including apocytochrome *b* and two subunits of the succinate dehydrogenase in *S. podlipaevi*; 18 subunits of the ATP synthase including mitochondrial DNA-encoded A6, six subunits of the cytochrome *c* oxidase, two subunits of the cytochrome *c* reductase and ten subunits of the succinate dehydrogenase, NDH2 and two components (E2 and E3) of the 2-oxoglutarate dehydrogenase complex in *N. esmeraldas* (Supplementary Table 2).

## Discussion

As was already mentioned above long-term cultivated trypanosomatids *L. tarentolae*, and *C. fasciculata* have lost ability to edit some of complex I subunits and do not possess active form of this enzyme (Sloof *et al*., 1994; Thiemann *et al*., 1994). It was shown for *T. brucei* that complex I is not essential for its bloodstream life form (Surve *et al*., 2012, 2017). Complex I contributes up to 20% of the electron flux of the respiratory chain in the procyclic form of *T. brucei* but it is also not essential and does not pump protons across the inner mitochondrial membrane (Verner *et al*., 2011). The main pathways of the electrons entry into the respiratory chain appear to be the complex II (Turrens, 1989; Denicola-Seoane *et al*., 1992) and/or the alternative enzyme NDH2 (Verner *et al*., 2013). Although some authors conclude that NDH2 is matrix-oriented (Surve *et al*., 2017), our previous results strongly suggest that NDH2 is oriented into the intermembrane space and therefore cannot regenerate NAD^+^ in the matrix (Verner *et al*., 2013). Within the mitochondria, mitochondrial NADH-dependent fumarate reductase, which converts fumarate to succinate, utilized by complex II, may have this function (Coustou *et al*., 2005). The only known exception to date was *P. serpens*, in which the complex I was demonstrated to be not only fully functional, but also the only proton pump in the respiratory chain (Nawathean and Maslov, 2000; Gonzalez-Halphen and Maslov, 2004; Čermáková *et al*., 2007). Our *in silico* analysis confirmed the presence of the genes encoding the complex I subunits and the alternative dehydrogenase NDH2 in the genomes of all analysed species (*B. ayalai*, *L. seymouri*, *N. esmeraldas* and *W. raviniae*). Most genes are present only in one copy, with the exception of *W. raviniae*, where some subunits are encoded by several genes. However, the mere presence of the genes encoding the complex I subunits is not equal to the functional enzymatic activity. *Leishmania tarentolae* and *C. fasciculata*, for example, also possess all the complex I subunit genes in their genomes, and yet their enzymes are not active because the subunits encoded by mitochondrial DNA are not edited (Sloof *et al*., 1994; Thiemann *et al*., 1994). Procyclic form *of T. brucei* has essentially no direct contribution of complex I to the mitochondrial membrane potential (Verner *et al*., 2011).

Significant differences in NADH dehydrogenase activity within the examined trypanosomatids confirm the statement that complex I is the most controversial enzyme of these parasites (Opperdoes and Michels, 2008; Duarte and Tomás, 2014). Regardless of the strong intensity of some bands, most of them were sensitive to 100 *μ*m DPI, similarly to the case of *T. brucei* (Verner *et al*., 2011, 2014). In addition to the expected resistance to DPI of the 2.2 MDa complex of *P. serpens* (Čermáková *et al*., 2007), we documented a similar phenomenon only in *N. esmeraldas* and *S. podlipaevi*. However, in contrast to *P. serpens*, these species have also the DPI-resistant activity in the range of about 1.3 MDa (Fig. 1B). It appears that this lower molecular weight complex is even more stable under conditions of native electrophoresis, as its activity was shown to be slightly stronger than that of the upper band under clear native conditions (Fig. 1A) and much stronger in the blue native gel (Fig. 1C). It also differs from the lower *P. serpens* bands (~600 kDa, DPI-sensitive) in our previous studies, which were suggested to be incomplete forms of the complex I (Čermáková *et al*., 2007; Verner *et al*., 2014).

It has been suggested that the 2-oxoglutarate dehydrogenase complex may be responsible for the detected NADH dehydrogenase activity in *T. brucei*, as up to four proteins of this enzyme were localized to the activity band (Panigrahi *et al*., 2008; Acestor *et al*., 2011). Our analysis revealed only one 2-oxoglutarate dehydrogenase subunit in *P. serpens*, two in *N. esmeraldas* and none in *S. podlipaevi* together with the complex I subunits. Therefore, we concluded that 2-oxoglutarate dehydrogenase does not contribute to the NADH dehydrogenase activity in the bands that we have analysed.

In this study, we detected a NADH dehydrogenase signal in the low molecular weight range (around 130 kDa) for the first time in trypanosomatids (*K. sorsogonicus* and *N. esmeraldas*). In the yeast *Yarrowia lipolytica*, the signal in the corresponding range comes from an alternative dehydrogenase (Čermáková *et al*., 2007). Our results confirm that this is also the case of *N. esmeraldas*, as we have detected the NDH2 protein in this area by LC-MS analysis. Interestingly, we have also identified NDH2 in the high molecular range along with the complex I subunits in this species. This could suggest that NDH2 functions in association with other proteins. Nevertheless, we revealed it with the complex I only in *N. esmeraldas*, but not in *P. serpens* or *S. podlipaevi*. We explain this discrepancy by either species-specific peculiarities, transient nature of this protein complex, or inconsistencies in databases used for downstream analysis. For example, we used proteome of the exact species *N. esmeraldas* for *Novymonas* but had to rely on data from *P. serpens* isolate Hart1 for the analysis of our model strain, 9T.

Spectrophotometric measurement of enzyme activities is more accurate and quantifiable than in-gel staining. Among the analysed trypanosomatids, sensitivity of the complex I activity to the low and high concentrations of rotenone has been previously documented only for *P. serpens* (Moyses and Barrabin, 2004; Čermáková *et al*., 2007) and *T. brucei* (Beattie and Howton, 1996; Fang *et al*., 2001), respectively. However, high concentrations of this inhibitor were shown to evoke non-specific effects (Hernandez and Turrens, 1998). It was later demonstrated that lower rotenone concentrations do not affect the NADH dehydrogenase activity of procyclic *T. brucei*, probably because the complex I is incomplete in this organism (Verner *et al*., 2011, 2014). In our experiments, rotenone inhibited the NADH dehydrogenase only in *P. serpens*. This can imply that none of the tested trypanosomatids have the *P. serpens*-like complex I. However, our experiments with capsaicin (which is another specific inhibitor of the complex I) led a different conclusion. The effect of capsaicin on NADH dehydrogenase activity in *P. serpens* was comparable to that of rotenone and inversely proportionally correlated with the effect of DPI in four other investigated species. Capsaicin was not effective in *K. sorsogonicus* and *W. raviniae*, while DPI inhibited their NADH dehydrogenase activity by 80% or more. The effects of DPI and capsaicin were similar in *N. esmeraldas*, *S. podlipaevi* and *P. serpens*. The resistance to rotenone in *N. esmeraldas* and *S. podlipaevi* may be explained by possible amino acid substitutions in NDUFS2, as has been described in other organisms, i.e. a substitution Tyr144Phe leads to 4× lower sensitivity to rotenone in *Y. lipolytica* (Tocilescu *et al*., 2010; Angerer *et al*., 2012). Taken together, our data strongly indicate the presence of a fully functional complex I in *N. esmeraldas* and *S. podlipaevi*.

MS analysis of the high molecular weight NADH dehydrogenase activity bands in *P. serpens* identified 32 subunits of the complex I (29 nuclear and 3 mitochondrial DNA-encoded) (Table 3). The total number of identified subunits is much closer to that of *Bos taurus* (45 subunits) (Carroll *et al*., 2006) or *Y. lipolytica* (42 subunits) (Abdrakhmanova *et al*., 2004) than to over 60 predicted subunits for trypanosomatids (Duarte and Tomás, 2014). Nevertheless, the complex I of *Y. lipolytica* migrates at about 880 kDa, which differs from the migration at over 2 MDa for *P. serpens* (Čermáková *et al*., 2007) and 1.3 MDa for *N. esmeraldas* and *S. podlipaevi* (this study). This can be explained by a higher number of the involved complex I subunits in trypanosomatids, or their significantly higher molecular weight. For example, the NDUFA6 subunit in most eukaryotes is about 15 kDa, whereas its predicted size in trypanosomatids varies from 77 to 83 kDa (Čermáková *et al*., 2007).

There could be several reasons why we did not detect all the complex I proteins in our analysis: (i) we used protein databases of the related species; (ii) we could not identify unique subunits, similarly to the case of trCOIV subunit of the complex IV (Maslov *et al*., 2002; Perez *et al*., 2014) and (iii) some predicted proteins were too short (Duarte and Tomás, 2014) or hydrophobic. A smaller number of subunits identified in *N. esmeraldas* and *S. podlipaevi* samples reflects the lower molecular weight form used for the MS analysis. This complex may be depleted of some weaker-bound subunits.

Importantly, we also detected several proteins of complex I encoded by mitochondrial DNA. This is the first experimental evidence for their existence in trypanosomatids. So far, only subunits of the complexes III, IV and V have been detected (Horváth *et al*., 2000*a*, 2000*b*, 2002; Acestor *et al*., 2011; Škodová-Sveráková *et al*., 2015*a*). We identified the ND8 subunit in three analysed species, ND1 in two and ND7 only in *S. podlipaevi*. We also detected the MURF2 – a mitochondrial protein of unknown function (Blum and Simpson, 1990). Its co-occurrence with other subunits of the complex I in all analysed species strongly suggests that it could be another subunit of this enzyme.

Comparison of bioenergetic metabolism in several trypanosomatid species suggests that these parasites have retained all the essential genes during evolution. Their expression depends on the specific living conditions – the availability of food and host–parasite relationships (Škodová-Sveráková *et al*., 2015*b*). Data obtained in this study indicate that the same rules apply to the complex I. Its loss is not only induced by the prolonged cultivation *in vitro*, but also may be influenced by natural conditions in different trypanosomatid species.

## Supplementary material

For supplementary material accompanying this paper visit https://doi.org/10.1017/S0031182020002425. click here to view supplementary material
