# Supplementary material for: Differences in mitochondrial NADH dehydrogenase activities in trypanosomatids
Source: Parasitology. 2021 Jan 7;148(10):1161–70. doi: 10.1017/S0031182020002425 (PMC8312217; doi:10.1017/S0031182020002425)
Supplement: Supplementary file 1 [file S0031182020002425sup001.zip › TAB 3.docx]

|  | *P. serpens* | *N. esmeraldas* | *S. podlipaevi* |
| --- | --- | --- | --- |
| N-module | NDUFV1 | NDUFV1 | NDUFV1 |
|  | NDUFA12 | NDUFA12 | NDUFA12 |
|  | NDUFV2 | NDUFV2 |  |
|  | NDUFA2 | NDUFA2 | NDUFA2 |
|  | NDUFS1 | NDUFS1 | NDUFS1 |
|  | NDUFA6 | NDUFA6 | NDUFA6 |
| Q-module | NDUFA5 | NDUFA5 | NDUFA5 |
|  | NDUFS7 | NDUFS7 | NDUFS7 |
|  | NDUFA9 |  | NDUFA9 |
|  | NDUFS8 (ND8*) | NDUFS8 (ND8*) | NDUFS8 (ND8*) |
|  |  |  | NDUFS2 (ND7*) |
| ND1-module | NDUFA8 | NDUFA8 | NDUFA8 |
|  | NDUFA13 | NDUFA13 | NDUFA13 |
|  | ND1* |  | ND1* |
| ND4-module | NDUFB11 | NDUFB11 | NDUFB11 |
|  | NDUFB10 | NDUFB10 | NDUFB10 |
|  | NDUFB1 | NDUFB1 |  |
| ND5-module | NDUFB7 | NDUFB7 |  |
|  |  | NDUFB9 | NDUFB9 |
| ACP | NDUFAB1 | NDUFAB1 | NDUFAB1 |
| Unique trypanosomatids  accesory subunits | NDUTB2 |  | NDUTB2 |
|  | NDUTB3 |  |  |
|  | NDUTB5 | NDUTB5 | NDUTB5 |
|  | NDUTB10 |  | NDUTB10 |
|  |  |  | NDUTB11 |
|  | NDUTB12 | NDUTB12 | NDUTB12 |
|  | NDUTB15 | NDUTB15 | NDUTB15 |
|  | NDUTB17 |  | NDUTB17 |
|  | NDUTB25 |  |  |
|  | NDUTB26 | NDUTB26 | NDUTB26 |
|  | NDUTB31 | NDUTB31 |  |
| Others | Tb927.10.5500 | Tb927.10.5500 | Tb927.10.5500 |
|  | Tb927.11.7212 |  |  |
|  | Tb927.11.15440 |  |  |
|  | MURF2* | MURF2* | MURF2* |
| Total | 32 | 24 | 27 |

**Table 3. Subunits of mitochondrial complex I detected by mass spectrometry analysis.**

Distribution of identified subunits to the modules of complex I is indicated in the left column. Designation of *H. sapiens* subunits in modules and ACP rows and *T.* *brucei* subunits in other rows were used. Subunits encoded by mitochondrial DNA are marked with * (ND1, ND7, ND8 and MURF2).
