# Supplementary material for: Differences in mitochondrial NADH dehydrogenase activities in trypanosomatids
Source: Parasitology. 2021 Jan 7;148(10):1161–70. doi: 10.1017/S0031182020002425 (PMC8312217; doi:10.1017/S0031182020002425)
Supplement: Supplementary file 1 [file S0031182020002425sup001.zip › TAB 2.docx]

| Species | Specific activity  [U/mg] | Inhibitor | Inhibition  % |
| --- | --- | --- | --- |
| *Phytomonas serpens* | 28 ± 11 | rotenone | 30 ± 4 |
|  |  | capsaicin | 42 ± 6 |
|  |  | DPI | 37 ± 4 |
| *Kentomonas sorsogonicus* | 39 ± 8 | rotenone | 2 ± 3 |
|  |  | capsaicin | 9 ± 8 |
|  |  | DPI | 100 ± 0 |
| *Novymonas esmeraldas* | 20 ± 9 | rotenone | 9 ± 7 |
|  |  | capsaicin | 34 ± 12 |
|  |  | DPI | 35 ± 8 |
| *Sergeia podlipaevi* | 27 ± 10 | rotenone | 6 ± 3 |
|  |  | capsaicin | 27 ± 9 |
|  |  | DPI | 20 ± 4 |
| *Wallacemonas raviniae* | 110 ± 24 | rotenone | 7 ± 4 |
|  |  | capsaicin | 8 ± 2 |
|  |  | DPI | 81 ± 13 |

**Table 2. Specific NADH dehydrogenase activity with and without inhibitors.**

NADH dehydrogenase activity was measured in the mitochondrial lysates of *P.* *serpens*, *K.* *sorsogonicus*, *N.* *esmeraldas*, *S.* *podlipaevi* and *W.* *raviniae* in the absence or presence of 10 μM rotenone, 300 μM capsaicin, and 100 μM DPI. Average values and SD of activities and their inhibition (in %) from at least 3 independent biological replicated (each measured in triplicates) are presented. One unit (U) of NADH dehydrogenase activity catalyses the oxidation of 1 nmol NADH per minute. Specific activity is calculated as U per mg of mitochondrial proteins.
