# Supplementary material for: Differences in mitochondrial NADH dehydrogenase activities in trypanosomatids
Source: Parasitology. 2021 Jan 7;148(10):1161–70. doi: 10.1017/S0031182020002425 (PMC8312217; doi:10.1017/S0031182020002425)
Supplement: Supplementary file 1 [file S0031182020002425sup001.zip › TAB 1.docx]

| species | *H. sapiens* | **T. brucei* | **B. ayalai* | **L. seymouri* | *N. esmeraldas* | *W. raviniae* |
| --- | --- | --- | --- | --- | --- | --- |
| membrane domain | NDUFB1 | Tb927.11.7390 | Baya_011_0530 | Lsey_0055_0260 | **+** | **+** |
|  | NDUFB7 | Tb927.9.11660 | Baya_100_0220 | Lsey_0192_0100 | **+** | **+** |
|  | NDUFB9 | Tb927.11.15810 | Baya_019_0320 | Lsey_0010_0080 | **+** | **+** |
|  | NDUFB10 | Tb927.11.9930 | Baya_039_0260 | Lsey_0091_0010 | **+** | **+ (2)** |
|  | NDUFB11 | Tb927.4.440 | Baya_165_0080 | Lsey_0525_0020 | **+** | **+** |
|  | NDUFAB1 | Tb927.3.860 | Baya_111_0040 | Lsey_0115_0040 | **+** | **+** |
|  | NDUFS5 | Tb927.3.5340 | Baya_092_0110 | Lsey_0041_0050 | **+** | **+** |
|  | NDUFA6 | Tb927.10.14860 | Baya_244_0010 | Lsey_0011_0010 | **+** | **+** |
|  | NDUFA8 | Tb927.10.12930 | Baya_093_0130 | Lsey_0013_0050 | **+** | **+ (3)** |
|  | NDUFA9 | Tb927.10.13620 | Baya_084_0070 | Lsey_0157_0100 | **+** | **+** |
| peripheral domain | NDUFA13 | Tb927.11.8910 | Baya_029_0060 | Lsey_0071_0190 | **+** | **+** |
|  | NDUFA12 | Tb927.9.12680 | Baya_004_0460 | Lsey_0186_0050 | **+** | **+ (2)** |
|  | NDUFA5 | Tb927.10.4130 | Baya_191_0090 | Lsey_0122_0100 | **+** | **+** |
|  | NDUFA2 | Tb927.11.16870 | Baya_038_0390 | Lsey_0241_0060 | **+** | **+** |
|  | NDUFS7 | Tb927.11.1320 | Baya_018_0020 | Lsey_0065_0230 | **+** | **+** |
|  | NDUFS6 | Tb927.6.4270 | Baya_060_0270 | Lsey_0209_0010 | **+** | **+** |
|  | NDUFS1 | Tb927.10.12540 | Baya_080_0190 | Lsey_0113_0150 | **+** | **+** |
|  | NDUFV2 | Tb927.7.6350 | Baya_155_0060 | Lsey_0197_0040 | **+** | **+** |
|  | NDUFV1 | Tb927.5.450 | Baya_008_1080 | Lsey_0248_0020 | **+** | **+** |
| NDH2 | - | Tb927.10.9440 | Baya_062_0020 | Lsey_0004_0940 | **+** | **+** |

**Table 1. *In silico* analysis of selected complex I genes and alternative dehydrogenase NDH2 encoded by nuclear DNA.**

All selected genes were detected in all analyzed trypanosomatid genomes. The table lists either the names of genes in the TriTrypDB that was used for *T.* *brucei*, *B.* *ayalai*, and *L.* *seymouri* or the „+“ sign indicating the presence in unannotated databases for *N.* *esmeraldas* and *W.* *raviniae*. All genes were found in one copy, except for a few genes of *W.* *raviniae*, for which a higher copy number is given in parentheses. Names of *H. sapiens* orthologues are also provided.
