# Supplementary material for: Differences in mitochondrial NADH dehydrogenase activities in trypanosomatids
Source: Parasitology. 2021 Jan 7;148(10):1161–70. doi: 10.1017/S0031182020002425 (PMC8312217; doi:10.1017/S0031182020002425)
Supplement: Supplementary file 1 [file S0031182020002425sup001.zip › Suppl Table 1.pdf]

**Suppl. Table 1. Nucleotide and amino acid sequences of proteins identified in the genomes of *Novymonas esmeraldas* and *Sergeia podlipaevi***

*Novymonas esmeraldas*

Membrane domain

**NDUFB1**

ATGGCGACAGCACAGGAGCTCGGTGCGGGCGGCATCGACGGGGCCGGCAAGCCC
ATCTTCGTGGCGACGGAGCACACACGCTTCTCGGCGAGAGAAGACCGTGAGCTCT
CCGACCAGGAGCAGCTGGCGTGGAAGTCCTTCCAGCTCTGGCGCACCTCCCTCTC
CATCGAGCGCCGCGACTCCGTGTACCAGACGATCACGAACGGCGACTACACCAA
CTTCAACCTCACACGACACATCATGGGCACCCCCTACCCTGTCATCGACAACACC
CCCTCCATCCTCAACACGGCCGCCGGCGTGCGCATGTACGAGCATGGCATCTCCT
TCCTTGTCGCCTACAGCTACAGCATGTGGGTGCGCTCCAAGGCGTCGCTGCGCCA
CGTCCACCTCCACCGCGCCACGCGCATGAGCGTGTCGTTTGGCACCTTCATGTTCA
CCGACTTGTGCTTCTGGTACCGCAGCATGTTCCGCCTCTCCGGCTTCCTGCCCAAC
GACTACGAGTGCCGCAAGTTCGGCGTGATGGAGTCGCAGGAGCGGCTGGAGCAG
AAGAAGGAGATGTGGGAGAAGTACTCCGCGTACAAGCGCGAGTGGTGCCGCCGC
TTCGACTACCACGTCTACGGCATCCGCCCCGGCGAGACGTTGAGCCTCTTCTCCG
CCTGCTGGCTCCCGGCGTGGGGCACGTCGTACGGCACCGTCACCGACTACCCGCT
GCGCAAGAACCCGTACTTCCTCAGCGCCACACCGCTGCGGGACATGTACACGGA
GTACTCCTTCACGATCGAGCCGCCGAGGTCCGCGAACTCGCCGCTGCCGCATGCG
CGGCCGGAGGTGTTGTACACCTTCCGTGGGCCGCGTGTCGCCTCCGATAGCAACA
AGCCGTGGATGGTGTGA

MATAQELGAGGIDGAGKPIFVATEHTRFSAREDRELSDQEQLAWKSFQLWRTSLSIE
RRDSVYQTITNGDYTNFNLTRHIMGTPYPVIDNTPSILNTAAGVRMYEHGISFLVAYS
YSMWVRSKASLRHVHLHRATRMSVSFGTFMFTDLCFWYRSMFRLSGFLPNDYECRK
FGVMESQERLEQKKEMWEKYSAYKREWCRRFDYHVYGIRPGETLSLFSACWLPAW
GTSYGTVTDYPLRKNPYFLSATPLRDMYTEYSFTIEPPRSANSPLPHARPEVLYTFRGP
RVASDSNKPWMV

**NDUFB7**

ATGACCGAGTACCTCGCCGATCTGGCCTCGGCCAAGCCGGGGGAGGCGATGCCG
CCCATCAACCTCGGGCCCTACGACAACCCCCTTCTCTGGAACAAGCTGGACCCCT
TTGGCGCCGATCGCGGCCACCAGCGCCGTCCGATGACGGTGTCGCGAGACTTCAT
GGAGCTGAACCAGGTGCCCATCGTCTATCGTGACCACTGCGTCCATCGCTGGATC
CCATTCCATCGCTGCATTCGCAACCTGAAGCCGGTCACGTGGGGCACGGTCAACT
GCCACGAGTTCGAGGAGTCGTGGATGGTGTGCCGCGCGCACGAGACGTACCGCC
TCCAGCTCCTCAAGAGCAAGTTTATGGCGCTCACGAAGGACTACACCGCGGAGG
ACAAGAAGTTCTTCCCTAGCATCCTCTACCTCGGCCTCCCCTACTACATGCCGTCC
TACTACTGGAACCTGGCCACGTCGCAGCGACTTAGCGGCTGGGACGAGAAGGAC
CCAGCGAACCCCGTCATGTGGCGCGAGCCCAACCGCTCCCTCATGCGCTCCGAGT

TCTCGCCCACCAACTGGGAGAAGGGCACGATGACCAGCGCCTACGGTCACAAGC
TCATCCCCGACGAGGTTGTGCACGACATGGTGCCGGGCTTTCCGCTGCCAGAGGA
CAAGCGTCCGCAGGCCGTGTGA

MTEYLADLASAKPGEAMPPINLGPYDNPLLWNKLDPFGADRGHQRRPMTVSRDFME
LNQVPIVYRDHCVHRWIPFHRCIRNLKPVTWGTVNCHEFEESWMVCRAHETYRLQL
LKSKFMALTKDYTAEDKKFFPSILYLGLPYYMPSYYWNLATSQRLSGWDEKDPANP
VMWREPNRSLMRSEFSPTNWEKGTMTSAYGHKLIPDEVVHDMVPGFPLPEDKRPQA
V

**NDUFB9**

ATGGTGGAGTACCATGAGTGGCACCTGCACGAGCCGCTGCTGCAGAACTACGAG
GGCTGGCGCATCCTCGACAACCCCAAGTACGAGCAGAAGTCAGACGCGTACGTC
TACGATTTCATGGGCAGCGTTCGCCACCTCGACCCGATCATCGACGACCCACGAC
TCACCCACAAGCAGCGTGTCTGCCGGCTCTACCGGTGGTCGCTCAAGGAGCTGCA
GATGTGGATGATCCAGCTAAGCGCGCACAAGCTGAACCTTGCCCACAAGGTGGT
GCGCCGCCGCTTCGAGAAGTACCGCTACGTCACCGACCCGGCCACGTGCGACATG
ATGGTGCGGCAGACACAGAAGTACCTCCGCGACAACGCCAACTTCCACTACCTGC
GCCGCAACAACGCTTCGCCGTGGAGCACGTACACCTTAGCAAATCCAATGTTCCA
CCCCGACAACTCGCTCGTCTACGACCACTGGACGCACTCGGAGGTGATGTGGTAC
GACGACGCCAAGCTGCATCGCTGGACGGCACACCACCCCATGTACGCCGGCCCTG
GTGAGGCGTCGGAGCGCTTCGGCGACATGGACGTGTCGCCCCACCTGCGCGGCAT
CACGTGGGCGATGGTGACGCTTCTTCTTTTGTGGTCCGCCTACCAAATCCTTGGCG
GCCCGGGTCTGCGCGGCGACCGACACTTCGAGGAGTGGACGCAGCAGTTCGATC
AGAAGCTCACCGGTGCCCTCTACGCCGAGGAGCGCAACTCCCGCTCCCGCAACAG
CGTTCTAGGCGGTGACTGGGACAGGGTGCTTGGCTTGGTCGGGATGCCGAAGGG
GTACTACCGCAACAACATCTCCGTCGTGGACAAGGCAAACTACCCGCCCGAGTAG

MVEYHEWHLHEPLLQNYEGWRILDNPKYEQKSDAYVYDFMGSVRHLDPIIDDPRLT
HKQRVCRLYRWSLKELQMWMIQLSAHKLNLAHKVVRRRFEKYRYVTDPATCDMM
VRQTQKYLRDNANFHYLRRNNASPWSTYTLANPMFHPDNSLVYDHWTHSEVMWY
DDAKLHRWTAHHPMYAGPGEASERFGDMDVSPHLRGITWAMVTLLLLWSAYQILG
GPGLRGDRHFEEWTQQFDQKLTGALYAEERNSRSRNSVLGGDWDRVLGLVGMPKG
YYRNNISVVDKANYPPE

**NDUFB10**

ATGACAGTGACCGCCGCGGCACACGGTGGTGACAGCCACGGGCACCACCACGAC
GACCACGACCACCACAGCGCCGGCGATGCCCCGCACGAGCGGATCGCCGCCCCG
TCCATGGCGTCGAAGGACTACCTCGACAACCTGCCCGCCTTCGACACGGCGAAGG
CGATGCCGCAGCCGCGCAGTGCCGTGGAGCGCTTCTCACGTGTGCTTCAGATGCA
GTTTGGGCAGGACCCCGACATGCCCACCCTCGGCCACAATGTCGAGCGCAAGTAC
CACAGCTTACTGGACTACCTCGCCAGTCCGATGGCTCCGCTGCGCCCCCGCACGT
ACGTGGACGCCTACACGCGTCCCAACCCGTGTCCGGAGGAGTACTGGTGGGCGTC
GTGGCGCTGGCCACGGACGAAGTACGTGAACGCGAAGGTGCCGCCGCCGGTGGA

TGGCAAGTACACGGACTACTACCACTACCTAGCGTTCAAGAACTGGCTGGAGCGG
GAGCGTGACGTGTACGTGGCGCACGCGAATCTCGTGCATGAGATGGTGTCACGCT
GTGTGGTGAAGGAGGGGCAGTACAACGCGGCCAAGAACTGCCGCCACCTCTACC
ACAAGGAGTTCGCCATGTCGCGTATGGAGGAGCTGAACCAGGCGTTGCTCTACAT
GGCCATGACAGGCAACGCAGCGATCCGTGAGACACCGTACCCGGAGAACTTCGT
GGAGGAGAAGCGAAAGATCTACGACGATTGGCTGTACCGCACACGCATGCGCAA
GCCGGGCGACGTGGCCTAG

MTVTAAAHGGDSHGHHHDDHDHHSAGDAPHERIAAPSMASKDYLDNLPAFDTAKA
MPQPRSAVERFSRVLQMQFGQDPDMPTLGHNVERKYHSLLDYLASPMAPLRPRTYV
DAYTRPNPCPEEYWWASWRWPRTKYVNAKVPPPVDGKYTDYYHYLAFKNWLERE
RDVYVAHANLVHEMVSRCVVKEGQYNAAKNCRHLYHKEFAMSRMEELNQALLYM
AMTGNAAIRETPYPENFVEEKRKIYDDWLYRTRMRKPGDVA

**NDUFB11**

ATGACGGGGTGGAATCAGAAGATGGCCAAGATGGCCTTTCGGCGGTCCAAGTTC
CGCCGCCTAGCCTGGGGTGACGGCGACCCGTACCCGTACACACCACGCGCCACGT
TCCGCTACCAGTGGGACGACTGGCCGATGTGGGAGAAGATGTGGCACCTCTGCGT
CGGAGTGCTCTGTGTCGAGTTCGCCGTGTGGGCCTACTACTCGAAGCTGAACAGC
CGAATGGACTGGGCCCGCGAGGAGGCGCTGCGTCGCATGCGTCTACGCCGTGAG
GCACAGGAGCTCATGATCCTGGAGGGCGAGACATTCGCGAGCGACATCCAGCCC
GACCGCCACTAA

MTGWNQKMAKMAFRRSKFRRLAWGDGDPYPYTPRATFRYQWDDWPMWEKMWH
LCVGVLCVEFAVWAYYSKLNSRMDWAREEALRRMRLRREAQELMILEGETFASDIQ
PDRH

**NDUFAB1**

ATGCAGCGCTCCATCGCTCGCCGCCTCGGCGCACGTGCGACCCTTCCCTTGACTG
CCGTCGGGATCAGGGGCTCGCAGCCTCAGGCCATCACACGTGCCAGCACTGCGAC
TGTCTGCGTGAGGGCTATCCCGTCCTCTCACTGCTCTGTACGCCTGTACTCCGGCG
GCCACCATGAGGAGCCTGCTGCTCGCAGTGGCCAGTACCTGCTAGACAAGAACG
ACGTCCTCACGCGTGTCTTGGAGGTAGTGAAGAACTTCGAGAAGGTGGACGCCTC
CAAGGTGAGCCCTGAGTCACACTTCGTGAACGACCTGGGCCTCAACTCCCTCGAT
GTAGTCGAAGTCGTCTTTGCCATTGAGCAGGAGTTCATCCTAGACATTCCCGATC
ACGACGCCGAGAAGATCCAGTCCATCCCAGACGCGGTCGAGTACATTGCCCAGA
ATCCGATGGCCAAGTAG

MQRSIARRLGARATLPLTAVGIRGSQPQAITRASTATVCVRAIPSSHCSVRLYSGGHH
EEPAARSGQYLLDKNDVLTRVLEVVKNFEKVDASKVSPESHFVNDLGLNSLDVVEV
VFAIEQEFILDIPDHDAEKIQSIPDAVEYIAQNPMAK

**NDUFS5**

ATGTCCTCCTACAGCGATGCTGATGTCGGCGCGCTGAACCGCGTCGTGGACGCCG
TGCGCCGCTGCCCCGGTGACATCCACCGTCCGGAGCTGGCGGCGCTGAAGGCGTG
GGCGGCCGACGCGGGCGCCACCTTCGCCCCCGCCCCCGCACCCGCCGAGGCCCCC
GCCCACGATGTGGATGACGAGAGCGACCCCGACGAGGAGCGCTGGACGCTGCAC
GATGCGGAGCCGGCGCCGATCGCAGAGAAGGCGGGCGAGCCCAGCGACGCCGAC
GCGGAGGCGGCCATGGCGGCCAAGGCGGAGGCGGCGGAGCTGCACAGTGATGGC
AAAGGCGCTGAGGCGCTGGCCAAGATGAGCGAGGCGCTGGCGCACAACCCTGGC
AGCGCCGCCTACTGGGGTCTGCGCGCCGTGTACCACCTCGAGGCCAACCTGCCAC
GTGCCGCGCTGCACGACGCCAACAAAGCGCTCGAGCGCAACCCGCAGAACGTCC
GCGCGCTGCGTGTGCGAGGGACGGTGAACCGCCACCTCGGTCGCTGGGAGGATG
CGCTGAAGGACCTCAGCGCCGCGCAGGCCATCGACTACGACGACGACACGAACC
CGATCCTTCGCTACGTCCAGGCCCGCGCCACGCAGCGCCACAAGCGTGCTCTGGC
GCGTCGACAGGCAGAGGAGGCGGAGGAGGAGGCGGCGGCGGCGCGGCGGCAGG
CGGAGCTTCGCCGCCAGCGCCAACAGGAGGCTGCTGAAGAGGAGGCGGAGGCGG
AGGCAGCGCGTGCAGCCGCAAGTGGGATGCCTGGCGGGATGCCTGGTGGGATGC
CTGGTGGGATGCCTGGTGGGATGCCGGGTGGGATGCCGGGTGGGATGCCGGGTG
GGATGCCTCCCGGTATGGAGGCGATCTTGCAGGATCCGGAGATCGTGGCGGCCAT
GCAGGACCCCGAGGTGGCGCCGAAGCTCGCGCAGATGATGCAGAACCCGATGGC
GGCGATGGGGATGATGAACGACCCCAAGGTGGGGCCCGTCGTACAGAAGATCAT
GTCGAAGATGATGGGCGGCGGCGGCTTCCCTGGTGCGGGCGGTGGCGGCATGCC
TCCTCGCGGTGGCGCCGCTGGCGGCGCGTCGCGCGCGGCGCCCACAGACGATCTG
GACTAG

MSSYSDADVGALNRVVDAVRRCPGDIHRPELAALKAWAADAGATFAPAPAPAEAP
AHDVDDESDPDEERWTLHDAEPAPIAEKAGEPSDADAEAAMAAKAEAAELHSDGK
GAEALAKMSEALAHNPGSAAYWGLRAVYHLEANLPRAALHDANKALERNPQNVR
ALRVRGTVNRHLGRWEDALKDLSAAQAIDYDDDTNPILRYVQARATQRHKRALAR
RQAEEAEEEAAAARRQAELRRQRQQEAAEEEAEAEAARAAASGMPGGMPGGMPGG
MPGGMPGGMPGGMPGGMPPGMEAILQDPEIVAAMQDPEVAPKLAQMMQNPMAA
MGMMNDPKVGPVVQKIMSKMMGGGGFPGAGGGGMPPRGGAAGGASRAAPTDDL
D

**NDUFA6**

ATGCTGCGACGCTTCTGTTCTCCGCTCGCTGTAGCGGCAACCGGGTTGCGCTTCAA
TGCCTCCAATGGGGGAACATTCGACGGAGGCGATGAGCCGATCCCCAACTCCACC
GGCAAGGACAATGCACCGGGTCTGTACAGCAAGAAGACCCCGGATGCGTACTTT
GAGGAACGGTTCCCTGACCGAAGCGGGGATGTGAAGTCGTTGAGGCCTCAGACG
TACGACGTGCCGCCAGGTCGATCGGACAACATCAAGTCGCCGGAGTTCAACACGT
CTCTAGGAAAGTTTGAGCAGGCACCCTATTTCACCGGTGGACCGCCAGCGATGCG
CTATCATGGCTACAAGCGTGAGTCTGCTAACAAAGAAGGAGTCGATTACCGCGAC
GTGCTGCCCAAGGTTCCCATGGAGGACCACCATCCGAACATGGATTTTCTCTCCG
TCACCGGGCGGCGTGAGGGAAGCACGTTTTTGCTTGCTAATGCCGGCGTGAACTG
GGAGTTGAAGGCGTCTGCGATGTCGCTCTATCGCACGATCCTCAAGGCGCTGCCA
ATGATCAAGCATTACTACTGGCTGCTGATCCCTCTTCCGCAGATGAAGGACAAGA

TCCGCTTCCGATTTCTGCAGAACCAGCACACCAAAGACCCGGACGCGATTCGCCA
TCTCCTTCACAACGGGTGGATGGAGTTCCAGGAGTCAATCATGTTTCGCCGCCCT
CGCGCGACCATCGAGAAGTACTTCGAATATGAGAGCATGGACACACTCATCGAG
CAGTACACAAGGGAGGAGGGTCTGATGAACCACGAGCGGGAGTTCTGGAACGGC
GAGGAGCAGCGGCGCGAGGGCCCGCACAATGGCCACTGGTCGTGGCTCGGGGAG
CAGTGCGAGCAGGAGTTTAGCAAGATCGCGGGCCGCATCCCCATGTCGTGGACC
ACCTCGAAGGGGTACTTCGAGAAGGGGCAGGCGGATGGCACGAACTACTGGGAG
AAGAACCTCGACTACGAGGGGTGGTACATCAAGAACGTTGACCCGGACCGTCAG
AACGCGCGGCGTGAGATGCAGGGCTGGGTGGAGAGCGGCTACAATCAGCCAAAG
CACTACGCTAGCAAGAACCGCCGCGGCTACCGCCGCATGGTGAAGGACATCGAG
ACGCTCATGGAAACCTCGATGGAGGACCTGTACACGCACAACCGCGAGCAGCTC
TTTCAGTACCTGATCCGTGAGACGAGCCCGGAGTCCAACCGCATCAACGCCGAGC
GCACGTTGGCGTACCAGGACGACGACTTCTACTCCACCCGCTTCGATGAGTACGA
GAAGTACGTGAAGCAGGCGATGCGTGAGATGCCGAACCCGCGCCTCTGGAAGAC
GGACGCGTTCTACTTCCGCCTTCGCTACCTCATCGCTCCGCTGGAGTACAACTGGG
CCAAGGTGCCGGTCGGCACCGCGCAAGAGAGGCTCTTCAATGAATGGATCTCAG
ACAACTGCAACTACGCCGTGTACACCAGCGACGCCTTCGCCCAGATCAAGGCGG
ACAAGATGCGCAATCCAATGGCGAGGACGTGGGCTGACTTCTACACGGACTTCG
ACCCCGACGTGCCGGACACGCGCCTCCTTCCATGGTACCACAAGGACTTCGACTA
CGACCGCCGCCACAAGTGGGACGAGCGGTGCATGCGCATGAAACGGTGGGTGCA
GGGCGGCACCATCGATGGCAAGCACGCTTTCTTCGACAGCATCATCGCCGAGTGG
GAGCAGTACGTGAACCGCCCGGAGCGGTTTCGCGCGCCGGACAACGCTGAGCGC
CGCTACGCGGCGCCGCGTATGGTGCAGCTCTACCGTGCACTGAACCGCGTGATGG
ATGTAGCGCTTGCGAATCAGATGAGGCAGGCGCTGGAGAAGGGCGGCGACTTTA
GCAAGCTCTCTGCGGAGGAGGTGCAGAAGCGCATCGCGGCGGCTGACTTCACGA
AGTTCCGGTTCGACGTCCCCGTTGTCATCTATCCTGACGGCCTAGTTCAGCCGCAG
CTTGGACTCGACGGCGGCAGCTCCAGCGGCACCGCTGCCACTGCTACTGCGACGG
CAGATGCGTAG

MLRRFCSPLAVAATGLRFNASNGGTFDGGDEPIPNSTGKDNAPGLYSKKTPDAYFEE
RFPDRSGDVKSLRPQTYDVPPGRSDNIKSPEFNTSLGKFEQAPYFTGGPPAMRYHGYK
RESANKEGVDYRDVLPKVPMEDHHPNMDFLSVTGRREGSTFLLANAGVNWELKAS
AMSLYRTILKALPMIKHYYWLLIPLPQMKDKIRFRFLQNQHTKDPDAIRHLLHNGWM
EFQESIMFRRPRATIEKYFEYESMDTLIEQYTREEGLMNHEREFWNGEEQRREGPHNG
HWSWLGEQCEQEFSKIAGRIPMSWTTSKGYFEKGQADGTNYWEKNLDYEGWYIKN
VDPDRQNARREMQGWVESGYNQPKHYASKNRRGYRRMVKDIETLMETSMEDLYT
HNREQLFQYLIRETSPESNRINAERTLAYQDDDFYSTRFDEYEKYVKQAMREMPNPR
LWKTDAFYFRLRYLIAPLEYNWAKVPVGTAQERLFNEWISDNCNYAVYTSDAFAQI
KADKMRNPMARTWADFYTDFDPDVPDTRLLPWYHKDFDYDRRHKWDERCMRMK
RWVQGGTIDGKHAFFDSIIAEWEQYVNRPERFRAPDNAERRYAAPRMVQLYRALNR
VMDVALANQMRQALEKGGDFSKLSAEEVQKRIAAADFTKFRFDVPVVIYPDGLVQP
QLGLDGGSSSGTAATATATADA

**NDUFA8**

ATGGACCAGTTCACGCAGCCGCTGGAAGGCATCTTCCGTGATGGCATTCCTGCGC
CGGTGCTGCGCGCCTTCGCCCCGCTCTTCCAAGCTCTGCCGAGCCTGCAAGAGCG
CGTCGAGGCGTCGCGCGACTGCTACTACTGGCGCGCCAACCCCATGAAGTGCTTG
GACGAGGACGTGAGCACCGTGAGCGGCTTCATGCAGGCCTCCGAGGCGAGCTTC
CGCCTGTGTCCCCAGCAGTCCGCGACGCTGCTCAAGTGCCACATGACAGAGCCGG
CACGCGCCGTGTACTTCTGCCGTGATGAGGAGTGGGAGTGGCGGAGCTGCTTGAT
GGATCAGACCGGCATCCGCTTCTGGCCCTACGCGAACGCGCCCATCGGCGCGCCG
TGGTCGAATGGCGGACAGACGGAGGACTTCCATCTCGAGGATCGCTTCTTCTACG
AGAACTTCTCCTTCTGGCGCCGTCGTGGGGCCATGCTGGCGGTGCGTGAGCGGGA
GCTCGAGGTCGCGGCGCAGCGCAAGCACTGGCTGGATGAGCAGACAGACGACAC
CTCCCTAGCGGCACCGAAGCCGACTCTAGCACCCATCGGCATCCACGCGAAGATC
AACTAG

MDQFTQPLEGIFRDGIPAPVLRAFAPLFQALPSLQERVEASRDCYYWRANPMKCLDE
DVSTVSGFMQASEASFRLCPQQSATLLKCHMTEPARAVYFCRDEEWEWRSCLMDQT
GIRFWPYANAPIGAPWSNGGQTEDFHLEDRFFYENFSFWRRRGAMLAVRERELEVA
AQRKHWLDEQTDDTSLAAPKPTLAPIGIHAKIN

**NDUFA9**

ATGCGGCGCTTCTCGGTGACGTCCACCGCGGCTGTCGCCGCTGCCGTTGGTGGTG
CGCGTGCCTTCTGGGACCCGTACGGACATCAGCCCGAGTCCATGTTCCTGGACCG
CAAGGACTTCAACCAGATGTACCCGACCACGAAGCCGAAGACCACTGGTGGCGG
CTTCGGCTACGAGCGCGGGCCGTACTGGGCCGCCATGCTGCTGCCCAACCCCGCC
GTTCGCCTTCCACACGAACGCCGCCGCCTCAACCCGAAGCCGGCGAAGCGCGTCA
CGGTCTTTGGCGCCAGCGGCTACCTCGGCGCTGAGGTGGTGCGGGAGCTGTGCGA
GCACCCCGACATCGAGACGGTGCGCGCCACCACGCGCTACCCCACACTCATCCCG
GAAGGATCGGACCTCGACGTGTTGCTGCGCCAGTACCCCGAGAAGCTAGAGCTG
CACGAGTGCGACGTGACGGACCGCATCCAGGTGAACGTAGCCGCCAACGGGAGC
GACACCCTCATCTTCGCGGTCGACTACCACGCCGAGTACGCCAACAACAGCCACC
ACGACGTCTTCCTGATCGGCGCCACCAACGTTAGCTGGACGGCCCGCAGCGTCCG
GGCAGAGCGTGTGATCTTCTGCAACGGACTCGACGCCACCTTCGCGTCGGAGTCG
AACTACGTCGACTTCCGCGCCCGCGGCGAGGACGCCGTCGGCGCCAACCACCCCG
ACGCCACCATCATCCGCTTCGGCCCGCTCTACGGCAGGAGGTACCGCTACCGCGG
CCTGGGCCGCTACATCTACCCCGCCTGCTTCCCCAACACGCAGGTGCAGCCGACG
TGGGTCGTGGACGCGGCGCGCGCGGTGGTGCGCTGCTCCATGTCGCACCGCGCGG
TGCGCTACAAGTTTGACCTCGGTGGCCCGCAGACGACGAGCCACGTCGAGGCGTT
CCGCGAGATCGCGCGGCACTTCGAGCAGCGCCTCGTCGTGCCGTGCTACCGCGGC
TTCGGCCGCTTCTTCGGCAAGCTCGCCCCCTGGACGGTGCCCAACCCCTGGTTCG
ACGACAACTACATCCTCACCTTCGAGCTGGACCAGGTGAACCGACGCAGCACCCT
CTTCGACCGCCTCGCGAGCTGGGACCGCATCGCCTACACGCCGCACTCCATCCGC
GAGGCGGCCGAGGTCGAGCGCGGCACCCGCACGCTGGCGCCGCTGCACGAGCTG
GACATCGCCTTCAAGAAGCTTGAGGCCGCCGACAAGGCCGCCTTCGAGCAGGAG
GAGGAGGCGGCGAAGAAGTACGGCATCCACCGCGCCAAGGCCGAGCCGGGCTTC
GGCCGCACCGACGGCCTCGAGGCGCTCGCGCAGGAGATCTACCCCGGCCAGCAG

TTCCGCATCAAGCCGCTCGAGGGCGCCAAGTACCCCTCCAACGTCGCTCAGCCCG
GCCCCACGGCGATCCAGTAG

MRRFSVTSTAAVAAAVGGARAFWDPYGHQPESMFLDRKDFNQMYPTTKPKTTGGG
FGYERGPYWAAMLLPNPAVRLPHERRRLNPKPAKRVTVFGASGYLGAEVVRELCEH
PDIETVRATTRYPTLIPEGSDLDVLLRQYPEKLELHECDVTDRIQVNVAANGSDTLIFA
VDYHAEYANNSHHDVFLIGATNVSWTARSVRAERVIFCNGLDATFASESNYVDFRA
RGEDAVGANHPDATIIRFGPLYGRRYRYRGLGRYIYPACFPNTQVQPTWVVDAARA
VVRCSMSHRAVRYKFDLGGPQTTSHVEAFREIARHFEQRLVVPCYRGFGRFFGKLAP
WTVPNPWFDDNYILTFELDQVNRRSTLFDRLASWDRIAYTPHSIREAAEVERGTRTL
APLHELDIAFKKLEAADKAAFEQEEEAAKKYGIHRAKAEPGFGRTDGLEALAQEIYP
GQQFRIKPLEGAKYPSNVAQPGPTAIQ

Peripheral domain

**NDUFA13**

ATGCTGCGCAGCACCGCACGTCGGCTGGTGCGGGACCCCGTTCCGACCGACACCA
AGGCGTTCTACACATGGTTCAGCGGCCAGGCCTACCGTCAGGAGCGCGTGATTCC
AGGCGGCTACCCCGCCGTTCGCGTGTACCCGGTGTACGGCAAGCGCTGGTTCACG
GGCCGCACCGTGGTGGCCCTCATCGCCGGCATCTCCATCTTCGGCGCGTGGATGC
GCCCGGAGCGCGAGCGCTACAACATGGAGATGATGATCGAATTCGCCGAGCGTC
AGTCCTCCTACTTGCCCTACCAGACCGCGGAGGTTCACCTGCGCTGCTTCGTCTCG
GGCTACAAGCGCCACCGCTTCGAGCAGGAGAACCTCATCGACAAGGGCTACGTC
GGCCTGACGTCCGAGTTCCGCAAGTTCTTCTACCACAGCGATGTGTGGCGTCCGC
CGCTGCACGACGTGCTCCTGCACCCCTACATCAAGTACGGTGGCCCCGTGGGTAG
CTACAACTGGTCTGTCGGCTACTTCTAA

MLRSTARRLVRDPVPTDTKAFYTWFSGQAYRQERVIPGGYPAVRVYPVYGKRWFTG
RTVVALIAGISIFGAWMRPERERYNMEMMIEFAERQSSYLPYQTAEVHLRCFVSGYK
RHRFEQENLIDKGYVGLTSEFRKFFYHSDVWRPPLHDVLLHPYIKYGGPVGSYNWSV
GYF

**NDUFA12**

ATGACGAGCGTCTTCACATTCGGCAGCCTCGGCGCCAACTCGATGGCGAAGAAGT
ACGGCGAGATGGCTGTCAGGTCGTCCAAGTGCGGGTCCAAGAACTTCGTCTACAC
CAAGTCGCACGACGACGCGCATAGCTTCCCGCGGCACCACCACCGCCAGGGCGA
CAACCCGGTGCGTCTCTCCTTCACCTTGGCCCGGTGGCGGTGGACGATGCACGAC
TTCCGCATGTTCGGCCTCTTCGGTGCACTGCGTAGACACTACTACATTGGCGAGG
CGTGGCGCCGCAAGGATGAGAAGATCTTCGTCGGCAAGGATGAGAACGGCAACA
AGTTCTGGCAGAGTCGTCGCGCTCAGGGCACCTTCTTCGTGCGCATAGTGGAGCC
GGCAGATCCCCACTGGTTCCGCGGCCAGTCGCCGCACGGCGCGAGCCCGATGTGG
ATGAAGTGGACGCAAGGCGGTGCCGCGCACACCCCGGCCCAGATGCAGGCACGC
GGTGAGTGGGGCCACAACTCGCGTCTTGGTATTCCCATGCCCTTCAACATCAAGT
ACAACGAGTGGTCGCCTCTCAACTCCTGCAACATTTACTCACGCGATCCCATGTG
GGTGGCCGCACCGGGCCTTCTCGTCAACCCCGAGCGGCGTGCTTTGCAGGAGGCG

GGCTTCTCGCGGTGGCTCTGGCAGAAGGGTCTCCCGGTGTACATGCCCTTCTGTG
GTGTCCACGACTACTCGGACGAGCTGGTGGAGGAGTTCTACCGCGGCCAGTGGGC
CTTCGGACGTGAGAGCAAGGGTAACGACCATGACGAGTGGCGTAACTAG

MTSVFTFGSLGANSMAKKYGEMAVRSSKCGSKNFVYTKSHDDAHSFPRHHHRQGD
NPVRLSFTLARWRWTMHDFRMFGLFGALRRHYYIGEAWRRKDEKIFVGKDENGNK
FWQSRRAQGTFFVRIVEPADPHWFRGQSPHGASPMWMKWTQGGAAHTPAQMQAR
GEWGHNSRLGIPMPFNIKYNEWSPLNSCNIYSRDPMWVAAPGLLVNPERRALQEAGF
SRWLWQKGLPVYMPFCGVHDYSDELVEEFYRGQWAFGRESKGNDHDEWRN

**NDUFA5**

ATGCTCGCCTCACGTCCCCTCCTGAGCGCGCTGCGTCGCACTGGGCGGCTTCTCAC
CATCAGCGATGAGTACATCCCTCGCGCCTTCCCGGTGAAGTCGACCACCGGCCTG
GCCGGTGTGGCGGTGGAGCCGCTGTGGAAGCCGAAGCTGCTCGCCGCCGCCTCG
GAGCTGCAGGCGTTCCTCCACACCTCCGACATCCCACCGGAGTCGACGTACTTCA
ACGTCACGATGACGCTGGTGAAGCGCATCAACTTTGGCGTGAAGGAGTGCCAGG
ATGATTGGTGCACGCTAGAGAAGAAGTACTTCTGGGGATGGCCTGTCGAGTACAT
CCTGCAGGTGACGTGGCGCGAGCTGGAGACGGCGCAGAAGTGGAACGAGTGGCG
CTTCTGGGAGCTGGACCCGGAGCAGGTGAAGCGCGTGGCCCGCGAGGACCAGGG
TATCGGCAAGGAGGGCATGGGCTACAACACCCCATGGGAGCAGGTCATCCGCGA
GGACTTCGACAAGCGCAAGAAGGCCCTGACGCAGGAGGAGATGGCGGAGCTGAA
GCGCATGGACACCGAGCGGATGGCGCGCGAGACGGCCGCGTACAAGGAGCGCAA
GGACCGCATCCGCGACGATCTAGAGAAGGCGCGTGGCGACATGCTGAAGAAGTT
CTTGAACAAGCGCTACGCCGTAGACAAGGACCTCATGCGAATGCAGCCCGGTAA
GAGCTACTCCGGCAAGAGTGCCGAGGACCTGATCGACGAGTTGCGCGCGTCTGTG
AAGCAGGGTCCTCCGGCGCCGTCCAAGTGA

MLASRPLLSALRRTGRLLTISDEYIPRAFPVKSTTGLAGVAVEPLWKPKLLAAASELQ
AFLHTSDIPPESTYFNVTMTLVKRINFGVKECQDDWCTLEKKYFWGWPVEYILQVT
WRELETAQKWNEWRFWELDPEQVKRVAREDQGIGKEGMGYNTPWEQVIREDFDKR
KKALTQEEMAELKRMDTERMARETAAYKERKDRIRDDLEKARGDMLKKFLNKRYA
VDKDLMRMQPGKSYSGKSAEDLIDELRASVKQGPPAPSK

**NDUFA2**

ATGTCGTGGCGCGCGCGGTTCACCCCATGCGTGGGCTCTCTCACGGTGTGGCTCA
ACCCCAAGGACCCGAACTGCTTTGGCGTCAGAAACTGGTGGCGCAACAATCTGCC
GGAGCTGCAGCTGCTCAACCCGTTCTGCACCTTCACGATTCAGGAGCTCTCCTTCG
GCGAGCCGCACATGTATGTGAACTACACGCCGACCGACCAGCGCATGATCCGCCT
CGCCGGTGCGACCGAGGAGGAGTGCGAGGAGATCATGGAGGCATGCATCACGTA
CGGCATGCATCACGCCATTCTCGAGCGCCCACGCACTGACGACGGCGGCGACTTG
GTGAACCAGCCTGCCATCACGTCCTTCGGCTACACGGAGAGCTTCACCGCAAAGC
TGGAGGTGGCGCCACCGGCGGACATCGGCCAGAAGACACCAGAGGGTGTCGACG
ACCCCGGCCAGAAGCCGCGCATGTTCCCCCGCAACGTAGGCTGCAAGTTGATGCC
GTAG

MSWRARFTPCVGSLTVWLNPKDPNCFGVRNWWRNNLPELQLLNPFCTFTIQELSFGE
PHMYVNYTPTDQRMIRLAGATEEECEEIMEACITYGMHHAILERPRTDDGGDLVNQP
AITSFGYTESFTAKLEVAPPADIGQKTPEGVDDPGQKPRMFPRNVGCKLMP

**NDUFS7**

ATGCTTCGTTTCACTCGTGCCTCCTTCACCGGCCGCGCGATGATCTCGCGCGGCAG
CCCCGAGTGGTCCCACCGCCTCGACCTCAAGAAGGGCAAGAAGACGACGCTGGC
GCACAAGCTTGGCACGAGCAAGCCAAACAACGCACTGCAGTACGCGCAAATGAC
GCTGCACGACTTGACGGAGTGGGTTGTTGCGTACTCGCCATGGCCGCTCACCTTC
GGTCTCGCGTGCTGCGCGGTGGAGATGATGCACTCCTACTCGTCACGCTATGACC
TTGACCGTTTCGGCATCGTTCCGCGTCCGTCGCCGCGCCAGGCGGAGATCATCAT
TGTGTCTGGCACTGTGACCAACAAGATGGCACCGCTGTTGCGCCACATTTACGTT
CAAATGGTAAACCCGAAGTGGGTCATCTCGATGGGAAGCTGTGCCAACGGCGGT
GGCTACTACCACTTCTCTTACGCGGTGCTGCGCGGGTGCGAGCGGTCGATCCCGG
TGGACTTCTGGATTCCCGGCTGCCCGCCATCTGCCGAGAGTCTCGTGTTTTGTCTC
CACAACCTGCAGAAGAAGATCCGCTGGCACGAGATCCAGAAGTACTCGGTGCGG
TGA

MLRFTRASFTGRAMISRGSPEWSHRLDLKKGKKTTLAHKLGTSKPNNALQYAQMTL
HDLTEWVVAYSPWPLTFGLACCAVEMMHSYSSRYDLDRFGIVPRPSPRQAEIIIVSGT
VTNKMAPLLRHIYVQMVNPKWVISMGSCANGGGYYHFSYAVLRGCERSIPVDFWIP
GCPPSAESLVFCLHNLQKKIRWHEIQKYSVR

**NDUFS6**

ATGAAGAAGACGTTCCCGTTACTGTGGACCTTCTCGGACAACCGAGGCTACTATG
GGCCCCCGGTCTACATGCCGTTAGAGTACGCCAGCCGCGTCACGAATCAGAAGCA
GCTGGTGTTCACCCATCCCAAGGATCCCAAGTACACGTGGAACACCGGCATCAAC
GAGCTCTCTAGTCTTCACCCCGGCATCCTCGGGCCGGGACGCAAGACGCCGCAGC
TGAACTACACTAGCCGCGGTGGCATCATCTGCGAAATCCCACCCGTGCCCGTCTA
CCGCGAGCACATCTGGTGCATGGGCCATGGCCACTTCACCCTGCAGCACCCACGT
ATCTTTATCAAATGCCCACGCAACAAGGTCGTCTGCTGCAAGTGGTGCCGTCTGA
AGTTCATCAACATGTCCACGGACGCCGACAACGACGACGACTGGTTCGAGGAGG
AGCAGAAGATTGCCACCACGCCGGAGACAGAGGAGGACCTGCGGCAGCCCATCC
GCGACATGACCGGCGTGCTGCGCGACAGCCCTTTCCAGGACGGCAAGGAGCCCG
ACCCGGATGTGTACCGCACCGTCTTCAACCCTGAGCGGTACCGCTGGAAGCACGC
CAACACAGACCACTACGAGGTGCACCCGGCCTACGCCAAGAAGGACGGCGATAC
TGAAGAGTGCGGCCATCACCACTAG

MKKTFPLLWTFSDNRGYYGPPVYMPLEYASRVTNQKQLVFTHPKDPKYTWNTGINE
LSSLHPGILGPGRKTPQLNYTSRGGIICEIPPVPVYREHIWCMGHGHFTLQHPRIFIKCP
RNKVVCCKWCRLKFINMSTDADNDDDWFEEEQKIATTPETEEDLRQPIRDMTGVLR
DSPFQDGKEPDPDVYRTVFNPERYRWKHANTDHYEVHPAYAKKDGDTEECGHHH

**NDUFS1**

ATGCCGACGCGTGCAGCAGCAGCAGCAGCAGCAGCAGCAGCAGCAGTGACGTTG
TCGTCGTCGTCGTCATCGCCGCTCTGGACGGCGCGTCGCCACTTCCGACCAGATG
GCACGCCGTACAACTCCGAGATGACTGAGATCGGACACGGCAGCACGTACCTGC
GCGGTGGGGCTGCCAACGCGGCGGCGGAGCAGGTGGCGGCGGGGCAGTACGCGG
AGAGCAAGCCCCGTGCCATCATGTTCGTCAACAAGCGGCCTGTCGAGATCATCCC
ACAGGAGGAGAACGTGTTGGAGGTGCTGGAGCGGGAGGGCATCACGGTGCCAAA
GTTCTGCTATCATCCGATCCTCTCCGTCGCGGGTAACTGCCGCATGTGCATGGTGC
AGGTGGACGGCACGCAGAACATCGTCGTGGCGTGCTCGACCGTGGCGCTGCCAG
GCATGTCCATCATCACGGACAGCCGCCTCGTGCGCGATGCACGCGAGGGCAACGT
GGAGCTCATCCTCATCAACCACCCGAACGACTGCCCCATCTGCGAGCAGGCGACG
AACTGCGACCTGCAGAACATCAGCATGAACTACGGCTCCGACATCCCACGCTACC
GCGAGGACAAGCAGGCCGTCGAGGACTTCTACTTCGACCCGCAGACGCGCGTCG
TGCTGAACCGGTGCATCCACTGCACTCGCTGCGTGCGCTTCTTGAACGAACACGC
GCAGGACTTCAACCTCGGACACATCGGCCGCGGTGGGCTCAGCGAGATCTCGACC
TTCCTCGATGAGCTGGAGGTGAAGACGGACAACAACATGCCGGTGTCGCAGCTGT
GCCCCGTTGGAAAGCTGTACCTCGGCGATGCGGATGAGAACGAAGACATCTTGC
GCGAACTCGAGGCGGTGGAGGCGGCCGCTGCCGCCACCGCCACCGCGTAG

MPTRAAAAAAAAAAAAVTLSSSSSSPLWTARRHFRPDGTPYNSEMTEIGHGSTYLRGG
AANAAAEQVAAGQYAESKPRAIMFVNKRPVEIIPQEENVLEVLEREGITVPKFCYHPI
LSVAGNCRMCMVQVDGTQNIVVACSTVALPGMSIITDSRLVRDAREGNVELILINHP
NDCPICEQATNCDLQNISMNYGSDIPRYREDKQAVEDFYFDPQTRVVLNRCIHCTRC
VRFLNEHAQDFNLGHIGRGGLSEISTFLDELEVKTDNNMPVSQLCPVGKLYLGDADE
NEDILRELEAVEAAAAATATA

**NDUFV2**

ATGCGCCATCAGAACACGGACTACGACAACACTCGCATCCCGTGGGACTTCACGA
CGGCGAGCTACGAGAAGATCCACAACGAGATCCTACCCAAGTTCCCACGCGGCA
AGCGCATCTCCGCCACCATCCCGCTGCTGCACCTCGCCCAGCAGCAGCAGGGCGG
GTACATCCCTGTGACGGCCATGTACAAGATCGCGAAGATATGCGAGGTGCCGCCG
ATGCACGTCTTCGAGACGGTGACCTTCTACTCCATGTTCAACCGCCACCCCGTGG
GCAAGTACCACATACAGTTCTGCCGTACCACCCCTTGCATGCTCTGCGGCGTCGA
CGAGCTCATGCACCGCACCATGCGGTACCTCAACGTGCGCATGCACGGCACCTCC
AGCGACGGTCTCATCACGGTGGGGGAGATGGAGTGCCTCGGCGCGTGCGTGAAT
GCACCGATGCTCGTCGTGAGTGACTACAGCGACCCACCCAACTTCTCCTACGACT
ATGTGGAGGACTTGACGTGGGACAGCGTCAAGACGCTCATCGAGAAGCTGCGCA
GTGGGCAGGCCTTCAAGATCGGGCCGCAGCGACCAGACCGCAAGTGCGCTGAGC
CGGCCGGCGGCCGCACGTCGCTGTTGTTCAAGGAGCCCCCAGGCCCATACTGCCG
TGACTTCGACGCCAAGCCGGAGGAGAAGAAGGCCGGAGCTGCACCAGCGAAGTA
G

MRHQNTDYDNTRIPWDFTTASYEKIHNEILPKFPRGKRISATIPLLHLAQQQQGGYIPV
TAMYKIAKICEVPPMHVFETVTFYSMFNRHPVGKYHIQFCRTTPCMLCGVDELMHRT
MRYLNVRMHGTSSDGLITVGEMECLGACVNAPMLVVSDYSDPPNFSYDYVEDLTW

DSVKTLIEKLRSGQAFKIGPQRPDRKCAEPAGGRTSLLFKEPPGPYCRDFDAKPEEKK
AGAAPAK

**NDUFV1**

ATGATGATGCGCCAGGGCCTTCTTCGCTCGTCCGCCGCGCTGCTGGATCGCGTGC
ACGGCCACCTCAAGGACCAGGATCGCATCTTCACGAACCTGTATGAGGACTTCGG
CACCGGCATCGCTGCGGCGGAGCGTCGCGGTGACTGGTACCGGACGAAGGACAT
CCTCCTCAAGGGCCATGACTGGGTGATCAACGAGATGAAGGCAAGCGGGCTGCG
CGGCCGCGGCGGCGCCGGCTTCCCGTCGGGGCTGAAGTGGTCCTTCATGCCCAAG
AAGAAGCCAGACGAGCGGCCGAGCTACCTCGTCGTCAACTGCGATGAGTCGGAG
CCCGGCACGTGTAAGGATCGAGAGATCATGCGCCACGAGCCGCACAAGCTGGTC
GAGGGGTCGCTCGTCGCCGGCTTCGCGATGCGCGCCCGCTACGGCTACATCTACA
TTCGCGGTGAGTTCTACAACGAGTGGCGCTCGGTGGAGCAGGCGATCCACGAGG
CCTACGAGAAGGGCTACCTTGGCAAGAACGCATGCGGCAGCGGCTGGGACTTCG
ACCTCTACACCTACCGCGGCGCCGGGGCGTACATTTGCGGGGAGGAGACGGCCA
TGATTTCCAGCATCGAGGGCGGGCAAGGGAAGCCGCGGCTGAAGCCGCCGTTCC
CCGCGAACGTCGGCCTCTACGGCTGCCCAACCACCGTGACGAACTGCGAGACGGT
GGCGGTGGCGCCGACGATCCTCCGCCGCGGGCCGCAGTGGTTCGCGCAGTTCGGG
CGGAAAGGCAACGCGGGCACGAAGCTGTACTCCATCTCCGGCCACGTGAACCGC
CCATGCACGGTGGAGGAGGAGATGAGCATGCCGCTGCGTGAGCTGATCGAGGTC
CACGCCGGCGGCGTTCGTGGCGGCTGGGACAACCTGCTCTGCGTCATCCCGGGCG
GCTCGTCGTGCCCGCTGATCCCAAAGCACGTCTGCGACAACATCTTGATGGACTA
CGACGCGCTCAAGGATGCGCAGACCGGGCTCGGCACGGCGGCGGTGATCGTCAT
GGACAAGTCGACCGACGTGATCGACGCGATCGAGCGGCTTTCGCAGTTCTACATG
CGTGAATCGTGCGGACAGTGCACGCCGTGCCGCGAGGGTGCGCCGTGGCTGGAC
AAGATGATGAAGCGCTTCGTCAACGGCAACGCCAAGAAGGAGGAGATCTACACC
ATGTGGGATGTGTCGAAGCAGATGGAGGGCCGCTCCATCTGCGCGCTCGGCACG
GCGGCGGCGTGGCCGGTGCAGGGACTCATCCGCCACTTCCAGCCGCTCATGGAGG
AGCGCATCGACCGCTTCTGGGAGGCGAACCCACACTGGGGAAAGGCCGGCTCGC
CGTGGCGCCGCTGGAAGACCCACCGGTACTACACCATGCAGAAGGGCGACCGTC
TCAACTGGGATGGCAAGATTGTGCGCAACTGGAACTAG

MMMRQGLLRSSAALLDRVHGHLKDQDRIFTNLYEDFGTGIAAAERRGDWYRTKDIL
LKGHDWVINEMKASGLRGRGGAGFPSGLKWSFMPKKKPDERPSYLVVNCDESEPGT
CKDREIMRHEPHKLVEGSLVAGFAMRARYGYIYIRGEFYNEWRSVEQAIHEAYEKG
YLGKNACGSGWDFDLYTYRGAGAYICGEETAMISSIEGGQGKPRLKPPFPANVGLYG
CPTTVTNCETVAVAPTILRRGPQWFAQFGRKGNAGTKLYSISGHVNRPCTVEEEMSM
PLRELIEVHAGGVRGGWDNLLCVIPGGSSCPLIPKHVCDNILMDYDALKDAQTGLGT
AAVIVMDKSTDVIDAIERLSQFYMRESCGQCTPCREGAPWLDKMMKRFVNGNAKKE
EIYTMWDVSKQMEGRSICALGTAAAWPVQGLIRHFQPLMEERIDRFWEANPHWGKA
GSPWRRWKTHRYYTMQKGDRLNWDGKIVRNWN

Alternative NADH dehydrogenase

**NDH2**

ATGCTGCGTCACACGGTGCTGCGGCTGACAAAGCCGAATGTGGTGGTGCTAGGCA
CGGGGTGGGCGGGCTGCTACGCCGCGCACCACCTCGACCCCAACCTGTGCAACAT
CCAGGTCATATCCACCCGCAATCACATGGTCTTCACCCCCCTGCTTCCCCAGACG
ACAACAGGCACGCTGGAGTTCCGCTCCGTGTGCGAGCCCATCACGAACATTCAGC
CCGCGCTCGCCAAGCTGCCACACCGCTTCTTCCGCAGCGTCATCTACGACGTCGA
CTTCGAGAAGAAGTTGGTGCAGTGTGTTGGCGTCGGTGTTGTGGGCGGCTCAGAG
AACGTCCCCGTCAGCACCTTCAGTGTCCAGTACGACTACCTCATCATGGCTCACG
GCGCGCGGCCCAACACGTTCAACATTCCTGGTGTGGAGGAAAAGGCGTTCTTCCT
GCGCGAGGTGAACGAGGCGCGCGGCATTCGCAAGCGCCTCGTGCAGAACATCAT
GGCTGCCGACCTGCCCACCACCCCTGTCGAGGAGGCGAAGCGGCTGCTGCACACC
GTCGTTGTCGGCGGTGGGCCGACCGGGATCGAGTTTGCCGCGAACCTCGCGGACT
TCTTCCGGGAGGATATCAAGAAGATCAACACCTCGCTGCTGCCCTACTGCAAGGT
GACTGTGCTCGAGGCGGGCGAGGTGCTGGGCTCCTTTGACACGGCGCTGCGACAC
TACGGCCAGCGGCGGCTGAGGCAGCTCGGTGTTGAGATCCGCAAGACTGCTGTG
GTCGGCGTCACAGACCAGGAGGTGTTCACCAAGTCTGGCGAGGTGTTGCCGACCG
GGCTGGTGGTGTGGAGCACCGGCGTCGGCCCGGGCCCGATCACGAAGGCGCTCA
AGTGCGACAAGACGAAGCGCGGGCGTGTCTCCATCGACAACCACCAGCGCGTCC
TCCGCGACGGCAAGCCGATCCCGAACGTGTTCGCGGTGGGGGACTGCGCAGCCG
AAAACGAGAAACCGCTGCCGACGCTGGCCGCCGTCGCCTCACGGCAGGGGCGCT
ACATCGGCAAGGAGGTGAACAACCTGCTCAAGGGCAAGACGATGAGCAAGCCCT
TCGTGTACCGCAGCCTCGGCAGCATGGCCTCCATCGGCAACCACTCCGCCATCGT
CTCCCTCGGGGAAAAGTACAAGATCGACGTGAACGGGTACGCTGCGTTGTGGGT
GTGGAAGAGTGCGTACTTGACGATCCTGGGCACCATCCGGAGCCGGCTCTACGTC
ATTGTCAACTGGGTGGGTAGTCAGGTTTTCGGCCGCGACATCACGTACATCGGCG
ACCTCAGCGAGGATCGCGTCTACGCTGCCTTGGCGGTGGAGGAGGTGTCGAAGG
AGGTGGGTCGCAAGAAGACGCACCAGATGCTGCACGCCCAGGACCCTAATAGCA
TCTTCACATCCGCCATGGCAGAAGAAGCGGCCAGGAAGGGCTTCCTGCCTCGCAA
GCTGGGCGAGCCAGCACCCGCGGCGCACCAGGCCCCCGCCGAGGACCCCTCCGC
GGCACCGGCCTCCAACGCTGCTGCGCCGACCACGCCAGAGAAGAAGGAGTAG

MLRHTVLRLTKPNVVVLGTGWAGCYAAHHLDPNLCNIQVISTRNHMVFTPLLPQTT
TGTLEFRSVCEPITNIQPALAKLPHRFFRSVIYDVDFEKKLVQCVGVGVVGGSENVPV
STFSVQYDYLIMAHGARPNTFNIPGVEEKAFFLREVNEARGIRKRLVQNIMAADLPTT
PVEEAKRLLHTVVVGGGPTGIEFAANLADFFREDIKKINTSLLPYCKVTVLEAGEVLG
SFDTALRHYGQRRLRQLGVEIRKTAVVGVTDQEVFTKSGEVLPTGLVVWSTGVGPG
PITKALKCDKTKRGRVSIDNHQRVLRDGKPIPNVFAVGDCAAENEKPLPTLAAVASR
QGRYIGKEVNNLLKGKTMSKPFVYRSLGSMASIGNHSAIVSLGEKYKIDVNGYAAL
WVWKSAYLTILGTIRSRLYVIVNWVGSQVFGRDITYIGDLSEDRVYAALAVEEVSKE
VGRKKTHQMLHAQDPNSIFTSAMAEEAARKGFLPRKLGEPAPAAHQAPAEDPSAAP
ASNAAAPTTPEKKE

*Wallacemonas raviniae*

Membrane domain

**NDUFB1**

ATGGCGGATGTCAAGGCTAATCAACTAGGCCCAGGTGGCGTGGATGCGGCGGGC
AAGCCCATCTTCATCTCCACTGAGCACACGCGCTACGCGCCTGCAAAGGACCGCG
AACTGACGGACCAAGAGCAGATCGCATGGAAGTCGTTCCAGCTCTGGCGCACGA
CACTGTCGATGGAGCGCCGCGACAACGTTTACAAGGGTATCGCGGATTCTGACTA
CACGAATTTCCACATGGCTTACCACATGATGGGCACACCGTACCCCGTCATTGAG
CCGGCGCCGAGCGTGTTCGAGACCTTCAAGTGCATCCGCTGGTACGAGCACGGCG
CGGCGGCTCTCGGTGGCTACGGCTATCTCACCTGGATCAACGCCAAGGCATCCAC
CCGCTACGCACGCATGTTCCAGACGTCACGCAACGCACTCAGCTTTGGCACTTTT
GCGCTTCTAGAGATCTGCTTTGCGTACCGTAGTATGTTTCGTCTTTCGGGCTACCT
GCCGAACGATCACGAGTGCCGCAAGTACGGCGTCATGGAGGACACCGCGCGCTT
GGAAAAGAAGAAGGAGATGTGGGAGAAGTATGCCGCCTACAAGAAGGAGTGGA
TGCGTCGGTTCGACTACCACGTCTACGGCATTCGCCCCGGAGACAACTGGAGCCT
GTTCTCTGCGTGCTGGTTCCCCGCCTGGTCGCCGTCCTACTGCAAGTCCACCGACT
ACCCACTCCGCAAGAACCCGCACTTCCTCACCGCTACCCCGCTCCGTGACATGTT
CGTCGACTCCAACTTCCACAACGAGATGGAGAAGAGCGACAACGTGCCGCTGGT
CCGTGCCAAGCCTGAGGTCAAGTACATCTACCATGGCCCTATCGACGCTCAAAAG
TCCACCAAGTAA

MADVKANQLGPGGVDAAGKPIFISTEHTRYAPAKDRELTDQEQIAWKSFQLWRTTLS
MERRDNVYKGIADSDYTNFHMAYHMMGTPYPVIEPAPSVFETFKCIRWYEHGAAAL
GGYGYLTWINAKASTRYARMFQTSRNALSFGTFALLEICFAYRSMFRLSGYLPNDHE
CRKYGVMEDTARLEKKKEMWEKYAAYKKEWMRRFDYHVYGIRPGDNWSLFSAC
WFPAWSPSYCKSTDYPLRKNPHFLTATPLRDMFVDSNFHNEMEKSDNVPLVRAKPE
VKYIYHGPIDAQKSTK

**NDUFB7**

ATGACGGAGTATCTGAAGGATCTTGCCTCCGCCGCGCCTGGCGAGCACCCTCCCA
TCAATCTTGGCCCGTACGATAATCCGCTTCTGTGGAACAAGCTTGATCCCTATGGT
GCTGATCGTGGTCACCAGCGTCGCCCCATGTCTGTGTCGCGCGACACGATGGAGC
TGCAGCAGGTGCCGATCGTGTTCCGTGATCAGTGCGTCCACCGCTGGATCCCGTT
TCACCGTTGCCTGAAGAATCTGAAGCCGGTCACGTGGGGTACCGTGAACTGCCAC
GAGTTCGAGGAGGCCTGGATGGTGTGCCGCTCGTACGAGACGTACCGCATGCAG
CTGCTCAAGAGCAAGTTCATGGAGATGACCAAGGACTACACCGCTGAGGACAAG
AAGTTCTTCCCTAGTGTCATGTACCTGGGAGTGCCGTACTACATGCCCACGTTTTA
CTGGTCCATGGCGGCGTCGCAGCGACTTAGTGGGTGGGACGAGAAGGACCCCCA
GAACCCGATGATGTGGAAGCAGCCCAACCGCTCGCTCATGCGCGCGGAGTTCTCG
CCGACAAACTGGGAGAAGGGCACCATGACCAGCGCCTACGGCCACAAGCTTATT
CCCGACGAGATTGTGCACGATATGGTGCCCGGCTTCCCACTGCCGGAGGACAAGC
GCCCTCAGGCCACTGCGTAG

MTEYLKDLASAAPGEHPPINLGPYDNPLLWNKLDPYGADRGHQRRPMSVSRDTMEL
QQVPIVFRDQCVHRWIPFHRCLKNLKPVTWGTVNCHEFEEAWMVCRSYETYRMQLL
KSKFMEMTKDYTAEDKKFFPSVMYLGVPYYMPTFYWSMAASQRLSGWDEKDPQNP
MMWKQPNRSLMRAEFSPTNWEKGTMTSAYGHKLIPDEIVHDMVPGFPLPEDKRPQA
TA

**NDUFB9**

ATGATGCGTCGTAGCTCTCTTCTGTTAAGCGGTGGCATGGTGGAGTACCACGAGT
GGCACCTGCACGAGCCGCTCCTGCAAAACTACGAGGGCTGGCGTATTCTCGACAA
CCCCAAGTACGAGCACAAGAGCGACGCCTACATCTACGATATCACCGGTAGCGTC
CGTCACCTAGACCCCATCATCGATGACCCTCGCCTGACCCACAAGCAGCGCGTGT
GCCGCCTGTACCGCTGGTCGCTGAAGGAGATTCAGATGTGGATGGTGCAGCTCAA
CGCGCACAAGTTCAACCTCGCATACAAGGTGATTCGCCGCCGCTTTGAAAAGTAC
CGCTACGTGTCCGACCCCGCCACGTGCGACATGATGGTGCGCCAGACCCAGAAGT
ACCTGCGCGAGAACGCCAACTTCCACTTTCTGCGCCGCAACAACGGCTCCCCGTG
GAGCACGTACACCCTCGCGAACCCCATGTTCCACCCCGACAACTCGCTCGTCTAC
GACCACTGGACGCACCCGGAGGTGATGTGGTACGACGACGCCAAGCTGCACCGC
TGGACCGCGCACCACCCCATGTACGCCGGTGCCGGCGAGATGTCCGACCGCTACG
GTGACATGGACGTCTCGCCCCATCTCCGTGCGATCACCTGGTGCATCTGCCTGCTC
GTGTTCGGCTTCGCCTTCTTCCAGCTCCTCGGTGGGCCACGCCTGATCATGCAGGG
TGGTGAGGACCCGCACTTTGAGAAGTGGTGCGCGAGCTTCGACCTCAACCTGGCC
GGCACGCTCTACGCGGAGGAGCGCAACACGCGTAGCCGCAGCAGCATGCTTGGC
TTCGACTGGGACCGCATCATGGGGCGCACTGGCGTCGAGAACGACGGCTTCCACT
TCTTCGATGTCAAGGCGGTGGACCCCAAGAACTACGCCTAA

MMRRSSLLLSGGMVEYHEWHLHEPLLQNYEGWRILDNPKYEHKSDAYIYDITGSVR
HLDPIIDDPRLTHKQRVCRLYRWSLKEIQMWMVQLNAHKFNLAYKVIRRRFEKYRY
VSDPATCDMMVRQTQKYLRENANFHFLRRNNGSPWSTYTLANPMFHPDNSLVYDH
WTHPEVMWYDDAKLHRWTAHHPMYAGAGEMSDRYGDMDVSPHLRAITWCICLLV
FGFAFFQLLGGPRLIMQGGEDPHFEKWCASFDLNLAGTLYAEERNTRSRSSMLGFDW
DRIMGRTGVENDGFHFFDVKAVDPKNYA

**NDUFB10**

ATGGGCGGCGACCACGGCGATCACGGTGATCACGGTCACGACGACCACCACCAC
GCCGCGGCCAAGTACGTTGACTACAAGGGCTCCACCCCGTATGAGAAGATCCCG
ATGCCAGCGATGGGCTCGCAGGACTACCTCGACAATCCTCCGGCCTTTGACACGA
GCAAGGCACCGGCTCCGGCCCGCTCGGCGGCGTCGCTTCACGGAGGCGCTGA
CGCTGCAGTGGGGTCAGGACCCCGACATGCCCACCCTCGGCAGCTCGCTCGAACG
CAAGTACAATGGCTTCCTGGATTACTTTCTTAGCCCCATCGCCCCGCTGCGGCCGC
GCACGTACGTGGACTCGCACACCCGCCCCAACCCGTGCCCTGAGGAGTACTGGTG
GGCGTCCTGGCGCTGGCCCCGCACCAAGTACGTCAACGCCAAGGTCCCACCGCCC
GTTGATGGCAAGTACTCAGACTACTACCACTACCTCTCCTTCAAGAACTGGCTGG
AGCGTGAGCGTGACGTGTACGTTGCGCAGGCCAACCTCGTCCATGAGATGCTCCA
GCGCTGTGTCGTGAAAGAGGGTCAGTACAACGCCGCCAAGAACTGCCGCCACTT

GTACAACAAGGAGTTCGCCATGTCGCGTATGGAGGAGTTCAATCAGGCGCTGCTG
TACATGGCCATGACGGGCAACGCCGCGATTCGCGAGACCCCGTACCCCGATGGCT
TCGTCGACGAGAAGCGCAAGATCTATGACGACTGGCTGTACCGCACGCGCATGC
AGAAGCCTGGCGATGTCGCGTAA

MGGDHGDHGDHGHDDHHHAAAKYVDYKGSTPYEKIPMPAMGSQDYLDNPPAFDT
SKAPAPARSAARRFTEALTLQWGQDPDMPTLGSSLERKYNGFLDYFLSPIAPLRPRTY
VDSHTRPNPCPEEYWWASWRWPRTKYVNAKVPPPVDGKYSDYYHYLSFKNWLERE
RDVYVAQANLVHEMLQRCVVKEGQYNAAKNCRHLYNKEFAMSRMEEFNQALLYM
AMTGNAAIRETPYPDGFVDEKRKIYDDWLYRTRMQKPGDVA

**NDUFB11**

GTAGGCAATCCACTCGCAAATGACCGGTTGGAATCAAAAGATGGCCAAGATGGC
CTTCCGGCGGTCCAAATTCCGCCGCCTAGCCTGGGGTGACGGTGACCCATATCCT
TACACGCCGCGCGCCACGTTCCGCTATCAGTGGGATGACTGGCCAATGTGGGAGA
AGGTGTGGCATCTGGCCGTCGGCATTCTCGGCATTGAATTCGGCATTTGGGCGTA
CTACTCCAAGCAAAACAGCCGCATGGACTGGGCCCGCGAGGAGGCTCTCCGTCGT
ATGCGTCTGCGCCGTGAAGCTCAGGAACTGATGATTCTCGAGGGTCAGACATTCA
ACTCCGATATTCAGCCCGATCGCCGTTAACAATCGCCAACAG

MTGWNQKMAKMAFRRSKFRRLAWGDGDPYPYTPRATFRYQWDDWPMWEKVWH
LAVGILGIEFGIWAYYSKQNSRMDWAREEALRRMRLRREAQELMILEGQTFNSDIQP
DRR

**NDUFAB1**

ATGCAACGCACTCCCATCTCTCGTATCTTTGTGCGCAGCCTCGCTGTCGGTGTTGG
TGCCTACAAGTGCGCACCTGCCCGCCCTGCGGTGCGTAATGCGTCGTGCGCTGTG
TCAGTGCCGTGCGCGTCCGTTCCCATGCGCTTCTACTCCGGCGGTGGCCACGAGG
AGCAGCACTCGCGTAGCGGCCAGTACCTTCTTGACCACAACGACGTGCTTACTCG
TGTCCTGGAGGTTGTGAAGAACTTCGAGAAGGTCGACGCGTCGAAGGTCACCCCC
GAGTCACACTTCGTGAACGACCTCGGCCTCAACTCCCTCGACGTCGTGGAGGTGG
TGTTTGCCATCGAGCAGGAGTTCATCTTAGACATCCCTGACCACGACGCCGAGAA
GATCCAGTCGATTCCTGACGCTGTGGAGTACATCGCGCAGAACCCGATGGCGAAG
TAA

MQRTPISRIFVRSLAVGVGAYKCAPARPAVRNASCAVSVPCASVPMRFYSGGGHEEQ
HSRSGQYLLDHNDVLTRVLEVVKNFEKVDASKVTPESHFVNDLGLNSLDVVEVVFAI
EQEFILDIPDHDAEKIQSIPDAVEYIAQNPMAK

**NDUFS5**

ATGCCGACGATCAGTGCGACCGACTCGGCTGCCGTGCAGCGCGTTGTCGACGCGC
TCAAGTCGTCCCCGCAGGAGATCCACGACCCGGCTCTGGCTTCACTCAAGGCGTG
GGCGGAGTCGTGCGGTGCGCACTTCGCCGCGGCCCCACACGCGGCGCCACACGCT

GCCCCACACGCGGCGCCAGAGGAGGAGGTCGCCTCCGAGGGCAGCGAACCCGAT
GAGGAGCGCTGGGATGTGGGCCAGCCACGCGAGGCGGCCGATGCGATCCCCGAC
AAAGACGGCGAGCCCAGCGACGAGGACACTGAGAAGGCCATGGCGCTCAAGGG
CGAGGCCGCTGATCTCAAGTCCGAGGGCAAGACCGAGGAGGCCATCGCGAAGCT
CGGTGAGGCTCTGCGCCTTGTGCCGCGCAACGCGATGTACTGGGGCCTGCGCAGC
GTGTACCAGCTCGAGGCCAAGCACCCCGCAGATGCCCTGCAGGACGCCAACCGC
GCTCTCACGTACAACCCGCAGAACGTGCGCGCTCTTCGCGTGCGCGGCACCGTCT
ACCGCCACCAGGCTCGCTGGGAGGACGCCCGCACCGACCTCAGCGCCGCGCAGG
CCATCGACTACGACGAGGCCACCGACGCCACACTCAAGTTCGTCCAGAGCCGCGT
TCACGCTCGCCAGCAGCGCACCAACAAGAAGAAGCTCGCTGCTGAGGAGGCAGC
GCACCAGCGTCAGGAGCAGCTCCGCAAGCAGCGCGAGGAGGAGATCCGCCAGGC
CGCGGCCGAGGCGCAGGGACGCTACGAGGCGGACGACGAAGAAGGGAACGGCA
TGTACGGTGGTATGCCCGGTGGCATGCCCGGTGGCATGCCCGGCGGTATGCCCGG
CGGTATGCCCGGGGGGATTCCGCCCGGCTTGGCTGCTGCGATGCAAGACCCTGAG
ATCATCGCCGCGATGCAGGACCCTGAGGTGGGCCCCAAACTGCAGCAGATGATG
AGCAACCCGATGATGGCCATGCAGTACATGAACGACCCCAAGGTTGGCCCCATC
ATGCAGAAGCTGATGGGCGCGATGATGGGAGGCGGTGGTATGCCCGGTGGTATG
CCCGGTGGTATGCCCGGTGGTATGCCCGGCGGTGCTGGTGGCGCGCCACCCAGGG
GCTTCCCCTCAGCGGGTGCGGGTGCGAAGGGTAACGATGACCTGGACTAG

MPTISATDSAAVQRVVDALKSSPQEIHDPALASLKAWAESCGAHFAAAPHAAPHAAP
HAAPEEEVASEGSEPDEERWDVGQPREAADAIPDKDGEPSDEDTEKAMALKGEAAD
LKSEGKTEEAIAKLGEALRLVPRNAMYWGLRSVYQLEAKHPADALQDANRALTYNP
QNVRALRVRGTVYRHQARWEDARTDLSAAQAIDYDEATDATLKFVQSRVHARQQR
TNKKKLAAEEAAHQRQEQLRKQREEEIRQAAAEAQGRYEADDEEGNGMYGGMPGG
MPGGMPGGMPGGMPGGIPPGLAAAMQDPEIIAAMQDPEVGPKLQQMMSNPMMAM
QYMNDPKVGPIMQKLMGAMMGGGGMPGGMPGGMPGGMPGGAGGAPPRGFPSAG
AGAKGNDDLD

**NDUFA6**

ATGCTGCGTCGCGTGATCGGAAAGAGCTCGTGCTCACTCCAGGTCCGCATGGTGG
CGTCAACCAGCGGCATTGGCACGTTCAACCCGAAGAAGGACTCCAAGGGAAACG
ACAACGACATGATCGGCCACCCGGAGAAGTCGTACGATGGCCCGATTAGAAACG
CCACTGGAATGGACAACCGTCCTGGCCTCTACGCCAAGAAACCGGAGGACACGT
ACTTTGAAAAGGCCTACCCTGACAAGATCGGTGAGCTGCCCAGCCACACTGTGAC
ATCGCCCAACGAGCCCAAATCGCGCGGTGACAACCTGAAAGTGCCCGAGTTCGA
CACCTCTCTTGGTAAGTTCGAGCAGGCGCCGTACATTTCTGGCGGCCCCAGCGCG
ATGCGGTACCAGAACTACCAGCGCGAACCACCAGTGGAAGGTGTGAACCTCAAC
GACACCGTCTTCGACCTCCCGTGGGATGATCACCACCCTAACTTCGAGTATGGCA
CCATCACCGGCAAGCGTGAGGGCAGCTCTCAAATGCTCGCCAACACCATCGCCGT
CTGGGAGTGCAACGCGGCAATCATGTCCCTCTACCGCGCCTGCCTCAAGTCACTC
CCCATGATCAAGCACTTTTACTGGCTTGTGACACCGCTGCCGCAGATGAAGGACA
AAGTGCGCCTGCGCTTTCTTCAAAATCAGCACGTCAAGGACCCCGACGCCATCCG
TCACCTGATCCACAACGGCTGGATGGAATATCAGGAGACGGTCATGTTCCGCCGC
CCGCGTGCCACCGTGGAAAAATACTTTGAGGCCGAAAGCATGGATGAACTGCTTC

GCCAGTATGTCAAGGAGGAGGGCCAGCGCAATGACGAGAGGGCCTTCTGGAACG
GTGAGGAGCAGCGCCGTGAGGGACCGTACGGCGGACACTGGTCGTGGCTTGGTG
AGCAGTCCGAGAAGGAATTCGAAAAGATTGCGGGGCGTGTGCCTGTGTCGTGGA
CGGCGTCCAAGGGCTACTTCGAAAAGGGCCAGGCCGATGGTACCAACTTTTGGG
AGAAAAATTTGGACTACGAAGGGTGGTACATCAAGAACGTCGACCCTGACCGCC
AGAACGCCCGTCGCGAAATGCAAGGCTGGGTGGAGAGCGGCTACAATCAACCAA
AGCACTACGCCAGCAAGAACCGCCGTGGCTACCGCCGAATGGTGAAGGACGTCG
AGACGCTCATGGAGACATCCATGGAGGACCTCTACACGCACAACCGTGAGCAGC
TCTTCCAGTACCTCATCCGTGAAACGCACCCCGAGTCCAATAGAATCAACGCGGA
GCGCACGCTTGCTCGCCAAGACGACGATTTCTACTCCACCAAGTTCGACGAGTAC
GAAAAGTACCTGAAGCAAACGATGCGCGAGATGCCGAACCCGCGCCTCTGGAAG
ACCGACGCCTTCTACTTCCGCCTCCGTTACTTGCTCGCCCCCTTAGAGTACAACTG
GGCAAAGGTGCCGATTGGCGTGGCACAGGAGAAGCTCTTCAACGAGTGGGTGTC
AGACAATGTCAACTACGCTGTATACACCAGCCCCGCATTCGCCGACATCAAGGCT
GACAAGGCACGCAACCCGATGGCAAAGACATGGGCAGACTTCTACACGGAGTTT
GACCCCGATGTCGCCGAGACACGCCGACTCCCTTGGTACCACCCTGAGTTCGACT
ACGACCGTCGCCACAAGTGGGACGAGCGCTGCATGCGCATGAAGCGCTGGGTGC
AGAGCGGGACCATCGACGGTAAGCTGCCATTTTTTGACAGCTTTGTCGCCGAGTG
GGAGCAGTACGTGAACCGCCCGGAGCGCTTCCGTGCTCCAGACAGTGCCGAGCG
GCGTTACGCAGCCCCGCGCATGGTGCAGCTGTACCGTGCGCTGAATCGCGTCATG
GACGTCGCGCTGGCGAACCAGATTGCCGAGACACTTTTGAAGGGTGCGAAGCGC
GAGGACCTGGCGAAGCTCTCTGTCGAGCAGATTCAAGAAAAGCTGGCGGCCGCC
GACCTTTCGTCCTTCAAGTTCGCTGTTCCCACCATCATCTACCCAGACGAGGCGGC
ACAACCACAGCTCGGACTCGATGGTCGTTCAACTGCAGCGAAGACAAAGGCCAG
TGCATAA

MLRRVIGKSSCSLQVRMVASTSGIGTFNPKKDSKGNDNDMIGHPEKSYDGPIRNATG
MDNRPGLYAKKPEDTYFEKAYPDKIGELPSHTVTSPNEPKSRGDNLKVPEFDTSLGKF
EQAPYISGGPSAMRYQNYQREPPVEGVNLNDTVFDLPWDDHHPNFEYGTITGKREGS
SQMLANTIAVWECNAAIMSLYRACLKSLPMIKHFYWLVTPLPQMKDKVRLRFLQNQ
HVKDPDAIRHLIHNGWMEYQETVMFRRPRATVEKYFEAESMDELLRQYVKEEGQRN
DERAFWNGEEQRREGPYGGHWSWLGEQSEKEFEKIAGRVPVSWTASKGYFEKGQA
DGTNFWEKNLDYEGWYIKNVDPDRQNARREMQGWVESGYNQPKHYASKNRRGYR
RMVKDVETLMETSMEDLYTHNREQLFQYLIRETHPESNRINAERTLARQDDDFYSTK
FDEYEKYLKQTMREMPNPRLWKTDAFYFRLRYLLAPLEYNWAKVPIGVAQEKLFNE
WVSDNVNYAVYTSPAFADIKADKARNPMAKTWADFYTEFDPDVAETRRLPWYHPE
FDYDRRHKWDERCMRMKRWVQSGTIDGKLPFFDSFVAEWEQYVNRPERFRAPDSA
ERRYAAPRMVQLYRALNRVMDVALANQIAETLLKGAKREDLAKLSVEQIQEKLAAA
DLSSFKFAVPTIIYPDEAAQPQLGLDGRSTAAKTKASA

**NDUFA8**

ATGGACCAGTTTGAGAAGCCGCTTGAAGGCATCTTCCGCGATGGCATCCCCGCGC
CTGTGTTGCGTGCGCTCGCCCCGCTCTACCAGGCGCTGCCCTCGCTGCAGGACAA
GATCACCACGTCGCGCGACTGCTACTACTGGCGCTCCAACCCGATGAAGTGCGTC
GACGAGGACGTCGACACCGTCACTGGCTTCATGCAAGCGTCGGAGGCCAGCTTCC

GCCTCTGCCCGCAGCAGTCTGCGACGCTGCTGAAGTGCCACATGACCGAGCCCGC
GCGCGCCGTCTTCTTCTGCCGCGACGAGGAGTGGGAGTGGCGTAGCTGCCTCATG
GACCAGACGGGTATCCGCTTCTGGCCGTACGCGAACGCCCCGATCGGCGCGCAGT
GGTCGAACGGCGGTCAAACCGAGGACTTCCACCTGGAGGATAGGTTCTTCTACGA
GAACTTCTCGTGGTGGCGCAAGAAGGCGTCGCTCATGGCAGTGCGCTCGCGCGAG
CTGGAGGTGCAGTCCGAGCGCAAGAACTGGCTTGATGAGAACGGTGATGGCTCG
ACGAATATGCAGGCACCGAAGCCCACGATTGCACCGGTTGCCATTGCCACCGCGC
GTGCCGTGAATTAG

MDQFEKPLEGIFRDGIPAPVLRALAPLYQALPSLQDKITTSRDCYYWRSNPMKCVDE
DVDTVTGFMQASEASFRLCPQQSATLLKCHMTEPARAVFFCRDEEWEWRSCLMDQT
GIRFWPYANAPIGAQWSNGGQTEDFHLEDRFFYENFSWWRKKASLMAVRSRELEVQ
SERKNWLDENGDGSTNMQAPKPTIAPVAIATARAVN

**NDUFA9**

ATGATGCTTCGCTTTGGTTCGAGGAGCGCGCAGGCGCTTGCCGTCGCTGAAGGCG
CCCGTACCTTCTGGGACCCGTACGGCCACCAGCCCGAGTCCATGTTCATGGACCG
CAAGGATCTCAACCAAATGTACCCCACCCAGAAGCCCAAGACCACTGGTGGTGG
CTTTGGTTATGAGCGTGGTCCGTACTGGGACGCGATGCTGCTGCCTAACCCGGCC
GTGCGTCTTCCGCACGAGCGCCGCCGTCTCAACCCCAAGGCGGCCAAGCGTGTGA
CGGTGTTCGGCGCGAGCGGTTACCTAGGCGCGGAGATTGTGCGCGAGCTGTGTGA
GCACCCTGACATCGAGAAGGTCCGTGCCACCACCCGCTACCCCACCCTGATCCCA
AAGGGCTCCGACCTGGACCTCCTGCTGCAGCAGTACCCCGAGAAGATTGAACTGC
ACGAGTGCGACGTGACGGACCGCATTCAGGTCAACGTGGCGTCCAACGGCAGCG
ACACGCTCATCTTCGCTATCGATTTCCACAACGAGTACGCGAATAATAGTCACCA
CGACGTCTTCCTGACCGGCGCGACAAACGTGAGCTGGACCGCCCGCAGCGTGCGT
GCGGAGCGCGTCATCTTCTGCAACGGCCTCGACGCGACGTTCGCCTCCGAGTCCA
ACTACGTTGATTTCCGCGCGCGCGGCGAGGATGCCGTTGGCGCGAACCACCCTGA
CGCCACCATTCTCCGCTTCGGCCCGCTGTACGGCAAGAACTACCGCTACCGCGGT
CTCGGCCGCTTCGTCTACCCGGCCTGCTTCCCCAACACGCAGGTGCAGCCGACCT
GGGTCGTGGACGCCGCACGCGCGGTTGTGCGTTGCTCCATGTCACAGCGCGCCGT
CCGCTACAAGTTCGACCTCGGCGGCCCGGAGACGTTCTCCCACGTCGAGTGCTTC
CGCGAGGTCGCGCGCAACTACGAGAACCGCCTGGTCGTTCCCTGCTACCGTGGCT
TCGGTCGCTTCTTCGGCAAGCTCGCCGGCTGGACGATCCCCAACCCGTGGTTCGA
CGACAACTACATCCTCACCTTTGAGCTCGATCAGGTCAACCGTCGCAGCACGCTG
TTTGACCGCCTCGCCAGCTGGGAGCGCATCGGCTACAAGCCGCACTCCATTCGTG
ACGCCGCGCGCGTGGAGCACGGCGAGGCCACACTCGCCCCTCTTCACGAGCTCGA
TACCGAGTTCAAGGCGATGGAGGCCGCCGACAAGGCTGCCTTTGAGCAGGAGGA
GGAGAACGCCAAGAAGTACGGCATTCACCGCGCCAAGGCGGAGCCTGGCTTTGG
CCGTTCCGATGGCCTGGAGGCGCTCGCGCAGGAGATCTACCCCGGCCAGCAGTTC
CGCATCAAGCCTCTCGAAGGCGCCAAGTACCCGTCCACTGTCAAGCACCCCGGTC
CGACCGCCATCCAATAA

MMLRFGSRSAQALAVAEGARTFWDPYGHQPESMFMDRKDLNQMYPTQKPKTTGG
GFGYERGPYWDAMLLPNPAVRLPHERRRLNPKAAKRVTVFGASGYLGAEIVRELCE
HPDIEKVRATTRYPTLIPKGSDLDLLLQQYPEKIELHECDVTDRIQVNVASNGSDTLIF

AIDFHNEYANNSHHDVFLTGATNVSWTARSVRAERVIFCNGLDATFASESNYVDFRA
RGEDAVGANHPDATILRFGPLYGKNYRYRGLGRFVYPACFPNTQVQPTWVVDAARA
VVRCSMSQRAVRYKFDLGGPETFSHVECFREVARNYENRLVVPCYRGFGRFFGKLA
GWTIPNPWFDDNYILTFELDQVNRRSTLFDRLASWERIGYKPHSIRDAARVEHGEATL
APLHELDTEFKAMEAADKAAFEQEEENAKKYGIHRAKAEPGFGRSDGLEALAQEIYP
GQQFRIKPLEGAKYPSTVKHPGPTAIQ

Peripheral domain

**NDUFA13**

ATGTTCCGCAACACTCGCGCGCGTCTTGTGCGGGACCCCGTGCCGGACGACACCA
AGGCGTTCTACTCGTGGTTTAGTGGCCAGGCATACCGCCAGGAGCGCGTTATTCC
GGGCGGCTATCCGGCTGTTCGTGTGTACCCGGTCTACGGCAAGCGCTGGATGACG
GGCCGCACGCTCATCGCTGTTATCGCAGGCGTCTCTCTCTTCGGCGCGTGGGTCCG
CCCCGAGAAGGAGCGCTACAACATGGAGATGCTTGTGGAGTTCTCAGAGCGCCA
GGCCGCGCACCTGCCGTATCAGCAGGCGGAGATGAACCTGCGTCTTCTGCTGTCT
GCGTACAAGCGTCACCGCTACGAGCAGGAGTGCCTTCTAGATAAGGGCTTTGTCG
GACTCACCAGTGAGTTCCGCAAGTTCTTCTACCACGACGATGTCTGGCGCCCACC
GCTGCACGACGTGCTCATGCACCCGTACCTCAAGTACGGTGGCCCCGTTACGAGC
TACAACTGGACCATTGCTTACTTCTAA

MFRNTRARLVRDPVPDDTKAFYSWFSGQAYRQERVIPGGYPAVRVYPVYGKRWMT
GRTLIAVIAGVSLFGAWVRPEKERYNMEMLVEFSERQAAHLPYQQAEMNLRLLLSA
YKRHRYEQECLLDKGFVGLTSEFRKFFYHDDVWRPPLHDVLMHPYLKYGGPVTSYN
WTIAYF

**NDUFA12**

ATGACTAGCATCTTCACGTACGGGTCGCACTGCGCGAACCCGCTCGCTGAAAAGT
ACGGACGCCTCGCGGTCAAGTCCTCCGCGTGCGGGTCGAAGAACTTCGTCTACCA
GAATCACTACGATGCGCGCATGACGTTTCCGCGTCACCACCACCGCGCTAGTGAT
AACCCGATCAGGGACTGCTTCACGTGGTCGCGCTGGCGCTGGACCATGCACGACT
TCCGCATGTTCGGCCTCTGGGGCTTCGTGAAGAAGCACGTCTACGTTGGTGAGAT
CTGGCGTCGCCGTGATGAGAAGATCTTCGTCGGCAAGGACGAGAACGGCAACAA
GTACTGGATGTCTCGCCGCACGCAGGGTACCAACAACGGCCGCTTCATTGAGCCG
AAGGACCCGCACTGGTTCCGCGGGCAGGCCCCTCACACGGCCAGCCCCATGTGGC
TCAAGTGGCTGCAGGGCAACTCGGCGCACACGCCTGCGCAGGTCAAGGCACGTG
GCGAGTGGGGTCTCAACTCGCGCATGGGTATGCCGCTGCCGTTCAACATCCACTA
CGACAACTGGGCACCGATCGCGTGGGGCGAGACGTGGTCGCGCGATCCGATGTG
GGTCTCCGCGCCGGGTATCCTCGTCAATCCGGAGCGCCGCGCACTCCAGGAGTCT
GGCTACTCGCGCTGGCTGCAGAACAAGGGCCAGCCGCTCTACATGCCCTTCTGTG
GCGTGCACGACTACCCGGATGAGCTCGTCGAGGAGTACTACCGCGGACAGTGGG
CCTTTGGCCGCACGAGCAAGGGTAATGACCACGACGAGTGGAGGAACTAA

MTSIFTYGSHCANPLAEKYGRLAVKSSACGSKNFVYQNHYDARMTFPRHHHRASDN
PIRDCFTWSRWRWTMHDFRMFGLWGFVKKHVYVGEIWRRRDEKIFVGKDENGNKY

WMSRRTQGTNNGRFIEPKDPHWFRGQAPHTASPMWLKWLQGNSAHTPAQVKARGE
WGLNSRMGMPLPFNIHYDNWAPIAWGETWSRDPMWVSAPGILVNPERRALQESGYS
RWLQNKGQPLYMPFCGVHDYPDELVEEYYRGQWAFGRTSKGNDHDEWRN

**NDUFA5**

ATGCTTGGTACGCGAAAGCTGCTGTCTGTGTTCCAGAGCACACTTGTTCTGCGAA
CGATTAGTGATGACTACATCCCCCGTGCGTTCCCGGTGAAGTCCACGACGGGTCT
GTCCGGTGTCGCTGTTGAGCCACTCTGGAAGCCCAAGCTACTCGCCGCGGCGTCG
GAGCTCCAGGCGTTCCTGGTGTCGTCGGACATCCCCACGGAGTCGACCTATTTCA
ACATCGCAATGACCTTAGTGAAGCGCATCAATTACGGCGTTCAGCAGTGCCAAGA
TGACTGGGCCACGCTAGAGAAGAAGTACTTCTGGGGCTGGCCTGTTGAGTACATC
CTCCAGGTCACCTGGCGAGAGTTGGAAACGGCGCAGAAGTGGAATGAGTGGCGC
TTCTGGGAGCTCGATCCGGAGCAGGTCAAGAAGGTCTCGCGCGAGGATCAGAAC
ATTGGCAAGGAGGGCCTTGGCTACAACACACCGTGGGAGCAAGTGGTGCGCGAG
GACTTTGACAAGCGCAAGAAGGCCCTCACACAGGAGGAGATGGCGGAGCTGAAG
CGGATGGACACGGAGCGGATGGCTCGCGAGACCGCCGCGTACAAGGAGCGCAAG
GACCGCATTCGTGATGATCTAGAAAAGGCCCGCGGCGACATGCTGAAAAAGTTC
CTGAACAAGCGCTTCGCTGTGGACAAGGATCTTATGCGCATGCAGCCGGGCAAG
GCGCAAAGCGGCAAGCATGCCGAAGACCTCATTGCCGAGCTTCGTGCGTCCGTGA
ATCAGAACCCCAGTGCCAAGGATGCACCTCCGAAGAAGTAA

MLGTRKLLSVFQSTLVLRTISDDYIPRAFPVKSTTGLSGVAVEPLWKPKLLAAASELQ
AFLVSSDIPTESTYFNIAMTLVKRINYGVQQCQDDWATLEKKYFWGWPVEYILQVT
WRELETAQKWNEWRFWELDPEQVKKVSREDQNIGKEGLGYNTPWEQVVREDFDKR
KKALTQEEMAELKRMDTERMARETAAYKERKDRIRDDLEKARGDMLKKFLNKRFA
VDKDLMRMQPGKAQSGKHAEDLIAELRASVNQNPSAKDAPPKK

**NDUFA2**

ATGTCCTGGCGGGCTCGCTTCACGCCGTGCGTGGGTTCTCTCACCGTCTGGCTCAA
CCCCAAGGACCCCAACTGCTTTGGTGTGCGCAACTGGTGGCGTAGCAACCTCCCT
GAGCTGCAGCTTCTCAACCCTTTCTGCACCTTCACAATCCAGGAGCTCTCCTTTGG
CGAGCCCCACATGTACGTGAACTATTCCCCCACTGACCAGCGCATGGTTCGCCTG
GCGGGCGCAACGGAGGAGGAGTGCGAGGACATCATGGAGGCGATCATCACCTAC
GGCATGAACCACGCCATCATCGAGCGTCCACGCACGGACGACGGTGGTGACCTC
ATCAACCAGCCCGCGATCACCTCGTTCGGCTACATGGAGAGCTTCACGGCCAAGC
TGGAGGTCGCCCCGCCTGCGGATATCGGCCAGAGGACGCCAGAGGGTGTCGATG
ATCCGGGGCAGCGCCCACGCGTCTGGCCGCGCAACGTTGGCTGCAAGCTCATGCC
GTAA

MSWRARFTPCVGSLTVWLNPKDPNCFGVRNWWRSNLPELQLLNPFCTFTIQELSFGE
PHMYVNYSPTDQRMVRLAGATEEECEDIMEAIITYGMNHAIIERPRTDDGGDLINQPA
ITSFGYMESFTAKLEVAPPADIGQRTPEGVDDPGQRPRVWPRNVGCKLMP

**NDUFS7**

ATGCTTCGCTTCACACGTCCGTCGCTCACGGGTCGTGCGATGATTTCTCGTGGTAG
CCCCGAGTGGTCGCACCGGCTGGACCTCAAGAAGGGTAAGAAGACCACGCTCTC
GCACAAGCTCGGTACGAGCAAGCCGAACAACGCGCTGCAGTACGCGCAGATGAC
CATTCACGATCTCACCGAGTGGGTGCTGACGTACTCACCCTGGCCACTCACCTTC
GGTCTCGCCTGCTGTGCCGTCGAAATGATGCACTGTTACGCCGCGCGTTACGACT
TGGATCGTTTCGGCATTGTGCCGCGTCCCACGCCGCGTCAGGCTGAAATCATCAT
TGTGTCTGGCACGGTCACGAACAAGATGGCCCCGCTGCTGCGCAACATTTACGTG
CAGATGGTGAACCCCAAGTGGGTGATTTCGATGGGCAGCTGTGCGAACGGTGGT
GGCTACTACCACTTTTCGTACTCCGTCCTTCGTGGTTGCGAGCGTTCCATTCCGGT
AGACTTCTGGATTCCCGGCTGCCCGCCGTCCGCCGAGAGCCTGGTGTTCTGCCTTC
ACAACCTGCAGAAGAAGATCCGCTGGCACGAGATTCAAAAGTACTCCGTCCGAT
AA

MLRFTRPSLTGRAMISRGSPEWSHRLDLKKGKKTTLSHKLGTSKPNNALQYAQMTIH
DLTEWVLTYSPWPLTFGLACCAVEMMHCYAARYDLDRFGIVPRPTPRQAEIIIVSGTV
TNKMAPLLRNIYVQMVNPKWVISMGSCANGGGYYHFSYSVLRGCERSIPVDFWIPG
CPPSAESLVFCLHNLQKKIRWHEIQKYSVR

**NDUFS6**

ATGAAGAAGACCAGTCTTCTTCGGTGGACCTTTTCCGATAACCGCGGTTACTACA
GTCCACCGATTTACATGCCCTTAGAGTACGCGAGTCGTGTGACCAATCAGAAGCC
CCTCCGCTTCACGCATCCTCGCGACCCCAAGTACGGCTGGAATACCCACGCGTAC
GAGCTGGCCAGCCTGCACCCCGGTCTGCTTGGCCCCGGCCGCAAGACGCCGCAGC
TGAACTTCTACAACCGTGGTGGCATCATCAACGAAATTCCCCCCGTCCCCGTGTA
CCGTGAGCACATTTGGTGCATGGGTCACGGCCACTTCACGCTGCAACACCCTCGC
ATTTTCATCAAGTGCCCCAAGGGTAAGGTTGTGGGCTGCAAGTGGTGCCGTCTCA
AATTTATCAATATGGCCACTGCCGAGGACAACGACGAGGACTGGTTTGAAGAGG
AGCACAAGATCGCCACGACGCCGGAGTCAAAGGAGGACTTGATGCAGCCGATTA
GGGACCTCGGAGGTGTCCTGCGTGATAGCCCATTCCAGATGGAAAGGAGCCTGA
CCCACACGTATACCGTGCTGTCTTCTCCCCTGATCGTCACCGCTGGAAGCACCCCC
ACACGAACCACTACGAAGTGCACCCGGCCTACGCTGGCAAGAAGTGCGAGCACC
ACCACTAAACCAAAGCGCACGAACGATACCACCAATGGTGGCTGTCTTGGGGTC
GTTCAAAGAAAAAAAAGAAAAGAAAAAAAAAGAAAAAAAACGTCAGAACGAAG
TCACATCACGCATGGTCTGCTGACGGCGTGGCACACAGTGCACACACGTGGGACA
AGGATGAAAGAGAGGCCTCCATTTACCCCTCTCGCAAAAAGAACCCGGCACAAA
CGCAAACCAGTGAACTCGTTAGTAGTATGTAGGAAATTGCTAGTACACAAACTGG
AGTCTCCCTGA

MKKTSLLRWTFSDNRGYYSPPIYMPLEYASRVTNQKPLRFTHPRDPKYGWNTHAYE
LASLHPGLLGPGRKTPQLNFYNRGGIINEIPPVPVYREHIWCMGHGHFTLQHPRIFIKC
PKGKVVGCKWCRLKFINMATAEDNDEDWFEEEHKIATTPESKEDLMQPIRDLGGVL
RDSPFQMERSLTHTYTVLSSPLIVTAGSTPTRTTTKCTRPTLARSASTTTKPKRTNDTT
NGGCLGVVQRKKRKEKKRKKTSERSHITHGLLTAWHTVHTRGTRMKERPPFTPLAK
RTRHKRKPVNSLVVCRKLLVHKLESP

**NDUFS1**

ATGTGCATGGTGCAGGTCGACGGCACGCAGAACCTCGTGGTGGCGTGCTCCACGG
TGGCCCTGCCCGGCATGTCCATCATCACGGAAAGCCGCCTGGTGCGCGACGCACG
TGAGGGCAACGTCGAGCTCATCCTCATCAACCACCCCAACGACTGCCCCATCTGC
GAGCAGGCCACGAACTGCGACCTCCAGAACGTGAGCATGAACTACGGCTCAGAC
ATCCCGCGCTACCGCGAGGATAAGAAGGCCGTCGAGGATTTCTACTTTGATCCGC
AGACCCGCGTCGTGCTCAACCGCTGCATTCACTGCACTCGCTGCGTGCGCTTCCTC
AATGAGCACGCGCAGGACTTCAATCTCGGCCACATTGGTCGCGGTGGCCTCTCGG
AGATCTCGACCTTCCTGGACGAGCTGGAGGTGAAGACGGACAACAACATGCCCG
TCTCTCAGCTGTGCCCCGTCGGCAACCTCTGCCTCGCCGACGCGGACGAGAACAA
CGAGATCCTGCGTGAGATTGAGGCCGCGGAGGCTGTGGCGGCCACTGCTGCGAC
CGCCTCCCATTGA

MCMVQVDGTQNLVVACSTVALPGMSIITESRLVRDAREGNVELILINHPNDCPICEQA
TNCDLQNVSMNYGSDIPRYREDKKAVEDFYFDPQTRVVLNRCIHCTRCVRFLNEHA
QDFNLGHIGRGGLSEISTFLDELEVKTDNNMPVSQLCPVGNLCLADADENNEILREIE
AAEAVAATAATASH

**NDUFV2**

ATGTGGCGTTTCCAACTCCCCGTCTCCGCGCGCCTGTCCGCGGTGGTGGCGGCCG
GTAGCAGCAGTGTGCTCACTGCCTCCCCCTCTCTAGCGCTGACGCAGCCCTGCCG
TGCTATCCACGGTGAGATGCGTCACACCAACACCGACTCCGACAACACGCGCATC
CCGTGGGACTTCACGACCGCGAGTTACGAGAAGATCCACCACCAGATTCTGCCCA
AGTTCCCGCGTGCTGAGCGTCGCTCCGCGGTGATCCCGCTCCTTCACCTGGCCCA
GCAGCAGCAGGGTGGCTACATCCCCGTCACGGCCATGTACAAGATCGCCAAGAT
CTGCGAGGTGCCGCCGATGCACGTGTTCGAGGTCGTGACCTTCTACTCCATGTTC
AACCGCCATCCGGTCGGTAAGTACCACATGCAGTTCTGCCGCACCACGCCGTGCA
TGCTGTGCGGCGGTGACGAGCTCATGGCGCAGACCCTGGCCTACCTCAACGTGGA
CATGCATGGCACGACCAGCGACGGCCTGATCACCGTCGGCGAGATGGAGTGCCT
CGGCGCGTGCGTGAACGCGCCGATGCTGGTCATTAGCGACTACAGCAACCCGCCG
AACTTCTCGTACGACTACGTCGAGGACGCCACCCTGGAGAGCCTCAAGGTCGTCA
TTGAGAACCTGCGCGCGGGCAAGCCGTTCAAGATTGGCTCGCAGCGCACGGACC
GCAAGTGGGCTGAGCCAGCGGGCGGCCGCACCTCCCTCTTCTTCAAGGAGCCGCC
GTCCCCGTACTGCCGCGACCTCGACGCGAAGCCTGAGGAGAAGAAGCCCGAGCC
ACCCAAGAAGTAA

MWRFQLPVSARLSAVVAAGSSSVLTASPSLALTQPCRAIHGEMRHTNTDSDNTRIPW
DFTTASYEKIHHQILPKFPRAERRSAVIPLLHLAQQQQGGYIPVTAMYKIAKICEVPPM
HVFEVVTFYSMFNRHPVGKYHMQFCRTTPCMLCGGDELMAQTLAYLNVDMHGTTS
DGLITVGEMECLGACVNAPMLVISDYSNPPNFSYDYVEDATLESLKVVIENLRAGKPF
KIGSQRTDRKWAEPAGGRTSLFFKEPPSPYCRDLDAKPEEKKPEPPKK

**NDUFV1**

ATGATGCGGAAGGCTGGTCTTCTGGCTACCTCGGGTATGCTTCTCGCGCTGAAGG
ACCAGGATCGCATTTTCACCAACCTGTACGACGACTTTGGGACTGACGTGGCGTC
GGCTGAGCGTCGCGGTGATTGGTACCGCACCAAGGACATCCTCCTCAAGGGCCAG
GACTGGATCATTAACGAGATCAAGGCCAGCGGTCTGCGTGGTCGCGGTGGTGCTG
GCTTCCCCTCCGGCCTCAAGTGGTCTTTTATGCCCAAGGTCAAGCCAGACGGCCG
CCCCAGCTACCTCGTCGTGAACTGCGATGAGTCCGAGCCCGGCACATGCAAGGAT
AGGGAGATTATGCGCCACGAGCCGCACAAGCTGGTCGAAGGCGCGCTCGTCGCC
GGCTTTGCCATGCGCGCGCGGTACGGCTACATCTACATCCGCGGCGAGTTCCACA
ACGAGCGCAAGGCTGTGGACAAGGCGATTCATGAGGCGTATCAGAAGGGCTACC
TGGGCAAGAACGCGTGCGGCAGCGGCTACGACTTCGACCTCTACACCTACGGCG
GCGCTGGTGCGTACATTTGCGGTGAGGAGACGGCGATGATTTCCAGCTTAGAGGG
CGGCCAGGGCAAGCCTCGCCTGAAGCCGCCGTTCCCAGCGAACGTGGGCTTGTAC
GGGTGCCCGACGACCGTCACCAACTGCGAGACGGTCTCTGTCGCACCGACGATTC
TGCGCCGCGGCCCTGCGTGGTTCGCGCAGTTTGGCCGCAAGGGCAACGCTGGCAC
AAAGCTCTACTCCTTCAGTGGCCACGTCAACCGCCCGTGCACGGTGGAGGACGAG
ATGAGCATGCCCCTGCGCGAGCTGATTGAGCGCCACGCGGGTGGCGTGCGTGGC
GGCTGGGACAACCTGCTGTGTGTGATTCCCGGTGGGTCCTCGTGCCCGCTGATCC
CCAAGCACATCTGCGACAATGTGCTCATGGACTACGACGCACTCAAGGCCGTCGA
AACGGGGCTGGGCACGGCGGCCGTGATCGTCATGGACAAGTCCACCGACGTCAT
CGCTGCCATTGAGCGTCTGTCCGTCTTCTACATGCGCGAGTCGTGCGGTCAGTGC
ACGCCGTGCCGCGAGGGCAGCCCGTGGCTGGACAAGATGATGAAGCGCTTTGTC
AACGGGAACGCCAAGAAGGAGGAGATCTACACCATGTGGGACGTCTCGAAGCAG
ATCGAGGGCCGCTCGATCTGTGCGCTTGGCACCGCCGCCGCCTGGCCCGTGCAGG
GCCTCATTCGCCACTTCACGCCCGTCCTCGAGGACCGTATCGAGCGGTACTGGGA
TGCGAACCCGCACTGGGGACAGTCGGGCTCCCCGTGGCGCCGATGGAAGACCCA
CCGTTACTACACGATGCAGAAGGGTGACCGCCTCAATTGGGATGGCAAGATCGTC
CGCAACTGGAACTAA

MMRKAGLLATSGMLLALKDQDRIFTNLYDDFGTDVASAERRGDWYRTKDILLKGQ
DWIINEIKASGLRGRGGAGFPSGLKWSFMPKVKPDGRPSYLVVNCDESEPGTCKDREI
MRHEPHKLVEGALVAGFAMRARYGYIYIRGEFHNERKAVDKAIHEAYQKGYLGKN
ACGSGYDFDLYTYGGAGAYICGEETAMISSLEGGQGKPRLKPPFPANVGLYGCPTTV
TNCETVSVAPTILRRGPAWFAQFGRKGNAGTKLYSFSGHVNRPCTVEDEMSMPLREL
IERHAGGVRGGWDNLLCVIPGGSSCPLIPKHICDNVLMDYDALKAVETGLGTAAVIV
MDKSTDVIAAIERLSVFYMRESCGQCTPCREGSPWLDKMMKRFVNGNAKKEEIYTM
WDVSKQIEGRSICALGTAAAWPVQGLIRHFTPVLEDRIERYWDANPHWGQSGSPWR
RWKTHRYYTMQKGDRLNWDGKIVRNWN

Alternative NADH dehydrogenase

**NDH2**

ATGCTTCGTGTCACTGCGCGTTTTTTGAGCAGGCCGAACGTGGTGGTGCTGGGCA
CGGGTTGGGCGGGCTGCTACGCAGCGCACAACCTGGATCCCTCGCTGTGCAACAT
TCAGGTGCTCTCGACCCGCAATCACATGGTTTTCACACCACTGCTCCCCCAGACC

ACCACCGGAACACTTGAGTTCCGCTCCGTCTGCGAGCCTGTCACAAACATTCAGC
CAGCGCTGGCACGTCTGCCGCATCGCTTCTACCGCAGCATGGTGTACGGTGTGGA
TTTCGACAACAAGGTGGTCAACTGTGTTGGCGTCGGTGTCGTCGGGGCGTCAGAG
AAGGTCCCCGTGCACACGTTCACTGTGTCGTACGACTATCTGGTGCTCGCGCACG
GCGCACGCCCGAACACGTTCAACATTCCAGGTGTCCAGGACAAGGCCTTCTTCCT
GCGGGAGGTGAACGAGGCGCGCGGCATTCGCAAGCGCCTGGTGCAAAACATTAT
GGCGGCCGATCTCCCCACCACGACCGTTGAGGAGACCAAGCGCCTTCTCCACTGC
GTCGTGGTCGGTGGTGGCCCGACGGGTATCGAGTTCGCCGCCAGTCTTGCTGAGT
TCTTCCTGGAGGACGTCAAGAAGGTGAACCGCAACCTGCTGCAGCACTGCAAGGT
GACCGTGCTGGAGGCTGGCGAGGTGCTCGGCTCGTTCGACGCCGCACTGCGCAAC
CACGGGCTGCGCCGCCTCAACGCGCTCGGTGTGGAGGTGAAGAAGAGCGCTGTC
GTGGGCGTGACGGAGAGCACGGTGCTGACCAAGGACGGGGAGACCCTGCCGTGT
GGTTTGGTGGTGTGGAGCACCGGTGTCGGCCCGTCCACCCTCACCAAGTCCCTCG
CCTGCGACAAGACCAAGCGCGGCCGTATCGCCATTGATGACCACCTGCGTGTCCT
GCGCGACGGGCAGCCCATCCCAGGCGTCTACGCAGGAGGTGACTGCGCCGCCAA
CGATGCGAATCCACTCCCGACACTCGCAGCTGTGGCGTCGCGCCACGGTATCTAC
CTCGGTCGCGAGCTGACCAAGATTCTTCGTGGTCGTGGGGAGAAGTGCAAGCCGT
TTGAGTACAAGAGCCTGGGCAGCATGGTCTCCATCGGAAACCGCAGTGCTCTCGT
GTCCCTCGGCAACACAACGCACATCGACCTGCACGGCCTTCTCGCCCTGTACTTTT
GGAAGTCAGCCTACCTCACCATTCTCGGCTCTGTCCGCAGCAAGCTCTACGTGTT
GGTAAACTGGTGCGGCAGCAAGATCTTCGGTCGCGACATCACGTACATTGGTGAC
CTCAGCGAGGACCGTATGTACAGCCTTCTCGCCGAAAACGAGGTGTCACGTGAGA
AGAACCGCCAGAAGGCGTACGAGACGCTTCGCACCATGTCCCCAGATGTTCACAT
CACTGCTGACAAGCTCGAGGAAAGTGCGCAACGCGGCTTCATTGAGCGCAAGGT
CACCCGTGACGACGCCAAGGCTGCAGCCCCTGCTGACGCCAAGAAGGTGGATGA
TGCAAAGAAGGTGGAGGACGCCAAGAAGGCGGACGCTGCCACCGCGCCCAAGCC
TGCTGCAACCGATAAGACACCAAAGGCTACGCCAGAAGAGGCAAAGACGGCGGA
CGCAAAGAAGTGA

MLRVTARFLSRPNVVVLGTGWAGCYAAHNLDPSLCNIQVLSTRNHMVFTPLLPQTT
TGTLEFRSVCEPVTNIQPALARLPHRFYRSMVYGVDFDNKVVNCVGVGVVGASEKV
PVHTFTVSYDYLVLAHGARPNTFNIPGVQDKAFFLREVNEARGIRKRLVQNIMAADL
PTTTVEETKRLLHCVVVGGGPTGIEFAASLAEFFLEDVKKVNRNLLQHCKVTVLEAG
EVLGSFDAALRNHGLRRLNALGVEVKKSAVVGVTESTVLTKDGETLPCGLVVWSTG
VGPSTLTKSLACDKTKRGRIAIDDHLRVLRDGQPIPGVYAGGDCAANDANPLPTLAA
VASRHGIYLGRELTKILRGRGEKCKPFEYKSLGSMVSIGNRSALVSLGNTTHIDLHGL
LALYFWKSAYLTILGSVRSKLYVLVNWCGSKIFGRDITYIGDLSEDRMYSLLAENEVS
REKNRQKAYETLRTMSPDVHITADKLEESAQRGFIERKVTRDDAKAAAPADAKKVD
DAKKVEDAKKADAATAPKPAATDKTPKATPEEAKTADAKK
